# Plasma proteins associated with circulating carotenoids in Nepalese school-aged children^☆^

**DOI:** 10.1016/j.abb.2018.03.025

**Published:** 2018-05-15

**Authors:** Abdulkerim Eroglu, Kerry J. Schulze, James Yager, Robert N. Cole, Parul Christian, Bareng A.S. Nonyane, Sun Eun Lee, Lee S.F. Wu, Subarna Khatry, John Groopman, Keith P. West

**Affiliations:** a Johns Hopkins Bloomberg School of Public Health, Baltimore, MD, USA; b Center for Human Nutrition, Department of International Health, Baltimore, MD, USA; c Department of Environmental Health and Engineering, Baltimore, MD, USA; d Johns Hopkins School of Medicine, Mass Spectrometry and Proteomics Facility, Baltimore, MD, USA; e Department of Biological Chemistry, Baltimore, MD, USA

**Keywords:** Proteomics, Plasma proteins, β-cryptoxanthin, Antioxidants, Carotenoids, Nepal

## Abstract

Carotenoids are naturally occurring pigments that function as vitamin A precursors, antioxidants, anti-inflammatory agents or biomarkers of recent vegetable and fruit intake, and are thus important for population health and nutritional assessment. An assay approach that measures proteins could be more technologically feasible than chromatography, thus enabling more frequent carotenoid status assessment. We explored associations between proteomic biomarkers and concentrations of 6 common dietary carotenoids (α-carotene, β-carotene, lutein/zeaxanthin, β-cryptoxanthin, and lycopene) in plasma from 500 6–8 year old Nepalese children. Samples were depleted of 6 high-abundance proteins. Plasma proteins were quantified using tandem mass spectrometry and expressed as relative abundance. Linear mixed effects models were used to determine the carotenoid:protein associations, accepting a false discovery rate of *q* < 0.10. We quantified 982 plasma proteins in >10% of all child samples. Among these, relative abundance of 4 were associated with β-carotene, 11 with lutein/zeaxanthin and 51 with β-cryptoxanthin. Carotenoid-associated proteins are notably involved in lipid and vitamin A transport, antioxidant function and anti-inflammatory processes. No protein biomarkers met criteria for association with α-carotene or lycopene. Plasma proteomics may offer an approach to assess functional biomarkers of carotenoid status, intake and biological function for public health application.

Original maternal micronutrient trial from which data were derived as a follow-up activity was registered at ClinicalTrials.gov: NCT00115271.

## Highlights

• Mass spectrometry-based proteomics and a novel statistical approach were combined to quantify protein relative abundances.
• We identified potential networks of interaction and candidate protein biomarkers of major circulating carotenoids.
• Plasma proteins could be measured in populations as functional biomarkers of carotenoid status and intake.

## 1 Introduction

Carotenoids are pigments occurring naturally in fruits and vegetables [1]. They are compounds synthesized from eight isoprenoid units, and more than 700 are found in nature [[2], [3], [4], [5], [6]]. Structurally, hydrocarbon carotenoids are referred to as carotenes and oxygenated carotenoids are termed xanthophylls. Among them, α-carotene, β-carotene, β-cryptoxanthin, lycopene, lutein, and zeaxanthin are the major dietary carotenoids found in human plasma [7]. While α-carotene, β-carotene and β-cryptoxanthin are provitamin A carotenoids, meaning they can be metabolized to retinol, lycopene, lutein and zeaxanthin cannot be converted to vitamin A [8]. Provitamin A carotenoids may be particularly important dietary sources for maintaining vitamin A status in impoverished regions, such as in rural Southern Asia [9], where vitamin A deficiency (VAD) has been shown to affect 20–35% of young children, school-aged adolescents and women of reproductive age [[10], [11], [12]] and is widely associated with low intakes of carotenoid-rich foods such as dark green leaves, yellow and orange vegetables and fruits and egg [12]. Carotenoids also appear to have *in vivo* antioxidant [13] and immunoregulatory [14] properties that are thought to give rise to frequent associations between their dietary intake or circulating concentrations and reduced risks of cardiovascular disease [15], various cancers [16] and macular degeneration [17,18]. Thus, plasma carotenoid concentrations may comprise a class of *in vivo* biomarkers that both reflect a diverse and nutritious diet [[19], [20], [21]] and nutritional, antioxidant, anti-inflammatory health of populations.

However, as molecules largely detected by chromatographic methods [22], carotenoids represent a group of micronutrients that are rarely assessed in low-middle income countries, signaling a need to explore novel approaches for their assessment in populations. In exploring plasma proteomics as an approach to ascertain potential biomarkers of micronutrient, functional, and health status in an undernourished population of school-aged children in Nepal, we have revealed associations between clusters of circulating proteins and micronutrient status (vitamins A, E, D and K, copper and selenium) [[23], [24], [25], [26]], inflammation [27], cognition [28] and anthropometry [29] in an undernourished population of school-aged children in Nepal. Findings to date suggest that plasma proteomics can identify proteins predictive of nutritional and health status that are candidate biomarkers with the potential to be measured by multi-analyte approaches for protein quantification. Missing from the emerging knowledge base is evidence of proteins that reflect plasma carotenoids, which could benefit from assays more readily conducted than conventional biochemical methods. The objective of this study was to explore the direction, strength and plausibility of association between plasma proteins and plasma carotenoid concentrations in a rural population of Nepalese school-aged children.

## 2 Methods

*Study cohort and field data collection.* We obtained plasma samples from 3305 children 6–8 years of age living in the southern plains district of Sarlahi, Nepal, born to mothers who had previously participated in a 5-arm antenatal micronutrient supplementation trial [30]. Following stratification by original maternal supplement allocation group, 1000 samples were randomly selected (200 per original trial group) from children with multiple aliquots of plasma samples, complete data records from both the original trial and current follow-up study, and valid birth size measures for multiple biochemical assessments [31]. Of these, 500 samples were randomly chosen for proteomics analysis, maintaining original trial balance. Children from whom samples were selected have been described in detail previously and are typical of children in the region [[23], [24], [25], [26], [27], [28], [29],^31^]. Follow-up data were collected on household socioeconomic characteristics, dietary frequencies and morbidity history for the previous 7 days and anthropometry (weight, height, mid-upper arm circumference), as reported earlier [24]. Weight-for-age Z-score (WAZ), height-for-age Zscore (HAZ) and body mass index (BMI)-for-age Z-score (BMIZ) were used to characterize nutritional status [32]. Venous blood was drawn from children following an overnight fast, which was processed into plasma aliquots and stored in liquid nitrogen in a field laboratory. Frozen plasma was transported in vapor phase liquid nitrogen shippers to the micronutrient analysis laboratory at Johns Hopkins University in Baltimore, Maryland, U.S.A. and stored at −80 °C until analysis. In both the original field trial and follow-up study, informed consents were obtained and protocols were approved by the Nepal Health Research Council in Kathmandu, Nepal, and the Institutional Review Board at Johns Hopkins Bloomberg School of Public Health in Baltimore, Maryland, USA.

*Plasma carotenoid analyses.* Plasma carotenoids including β-carotene, lutein and zeaxanthin, β-cryptoxanthin, α-carotene, and lycopene were analyzed by HPLC (Waters 2795) with a quaternary gradient pump, autosampler, photodiode array detector and Empower 2 software following the procedure of Yamini et al. [33]. The peaks of lutein and zeaxanthin could not be distinguished as they are combined. Separation of carotenoids was achieved using an All sphere ODS-2, 5-μm, 4.6-mm column (Alltech) and a Supelguard Discovery C18 2-cm × 4.0-mm guard column (Sigma-Aldrich). The assay was calibrated using the National Institute of Standards and Technology standard reference material SRM968d.

*Plasma proteomics analysis.* Mass spectrometric and proteomics procedures developed for this study have been reported elsewhere [23,34]. In brief, 500 plasma samples (40 μL) were immunoaffinity-depleted of six high abundance proteins (albumin, IgG, IgA, transferrin, haptoglobin and antitrypsin), which constitute 85–90% of total plasma protein content, using a Human-6 Multiple Affinity Removal System (MARS) LC column (Agilent Technologies). Protein extracts (100 μg each) were TCA/acetone precipitated, trypsin digested, labeled by isobaric mass tags (iTRAQ 8-plex reagents), and then seven samples plus one pooled sample for quality control was fractionated by strong cation exchange (SCX) chromatography and analyzed on a LTQ Orbitrap Velos mass spectrometer (Thermo Scientific). MS/MS spectra were searched against the RefSeq 40 database using Mascot (Matrix Science) through Proteome Discoverer software (version 1.3, Thermo Scientific) to quantify proteins with respect to the within-iTRAQ medians of log_2_ transformed and normalized reporter ion intensities derived from Proteome Discoverer. Data were obtained from 72 iTRAQ experiments with average 589 ± 65 proteins quantified per iTRAQ experiment. A total of 4705 proteins were detected, with 982 quantified in >10% (n > 50) of all samples [23] and 146 proteins measured in all 500 samples, representing the plasma proteome for this study.

### 2.1 Statistical analysis

Detailed information on estimation of protein relative abundance from reporter ion intensities within each iTRAQ experiment was published elsewhere [34]. We applied linear mixed effects models (LME) to determine the association between log_2_ transformed plasma concentration of each carotenoid and the relative abundance of individual plasma proteins accounting for multiple iTRAQ experiments.

The expected values of log_2_ carotenoid concentrations for each individual protein from the LME can be expressed as

E{N_rk_} = b_0_ + B_r_ + b_1_ P_rk_

where *N*_*rk*_ is the log_2_-transformed plasma concentration of each carotenoid, *k* is the index for each sample in each *r* iTRAQ experiment, and *P*_*rk*_ is the protein relative abundance estimate. The parameter *b*_*0*_ is the estimate of the intercept which is the overall mean concentration of each carotenoid; *B*_*r*_ is the random deviation of experiment r from this mean; and, *b*_*1*_ is the estimate of the slope of the nutrient:protein association. Statistical significance of a protein:nutrient association was assessed by a two-sided hypothesis test for *b*_*1*_ = 0. For individual significant nutrient:protein correlations, a *q*-value, an adjusted *p*-value to control false discovery rate (FDR) was reported [35]. Protein:nutrient correlation (*r*) and R^2^ were calculated based on the observed plasma carotenoid concentrations and their respective best linear unbiased predictions from the LME models [36].

We present a list of all proteins with an FDR less than 10% (*q* < 0.10) in their associations with each plasma carotenoid, and their corresponding Human Genome Organization (HUGO) gene symbols [37], the number of samples with detected protein values (n), protein:nutrient correlation (*r*), the amount of variance in nutrient concentration as explained by the protein (*R*^*2*^), *p*-value derived from testing the fixed effects slope of carotenoid concentration on protein abundance, chance-adjusted *p*-value (*q*), the slope (*b*_*1*_), denoting relative (%) change in carotenoids per 2-fold (100%) increase in relative abundance of each protein and GenInfo identifier (*gi*) accession number [37] is provided in the tables.

Datasets of plasma carotenoid concentrations and protein relative abundance presented in this study are available in Supplementary Table 1. All analyses were conducted using open source software built under the R statistical computing environment [38].

## 3 Results

Nutritional status and demographic characteristics of study children (n = 500) are shown in Supplementary Table 2. Study children were undernourished as reflected by low anthropometry Z-scores: 48.5%, 39.1%, and 16.1% of them were considered underweight, stunted, and thin (weight-for-age, height-for-age, and BMI-for-age Z-scores < -2, respectively), relative to the World Health Organization (WHO) reference population [32]. For β-carotene, 41.6% children had plasma concentrations <0.09 μmol/L (Table 1), which is considered low [33]. While the plasma xanthophyll carotenoids, β-cryptoxanthin and lutein/zeaxanthin were detected among all children, plasma carotenes, α-carotene and lycopene, were more likely to be below detection limits. Values below the detection limit were not included in the distribution of values shown in Table 1.

**Table 1:** Plasma carotenoid concentrations (μmol/L) of children, 6–8 years of age, Sarlahi, Nepal.

Plasma carotenoids	n^a^	Median (IQR)^b^
β-Carotene^c^	497	0.10 (0.06, 0.19)
Lutein/zeaxanthin	500	0.34 (0.25, 0.48)
β-Cryptoxanthin	500	0.06 (0.04, 0.12)
α-Carotene	481	0.01 (0.01, 0.02)
Lycopene	171	0.02 (0.01, 0.03)

a n represents the number of samples with detectable concentration of each carotenoid.

b IQR = interquartile range.

c Cutoff for low plasma β-carotene is 0.09 μmol/L, below which 207 values were observed (41.6%).

**Fig. 1:**
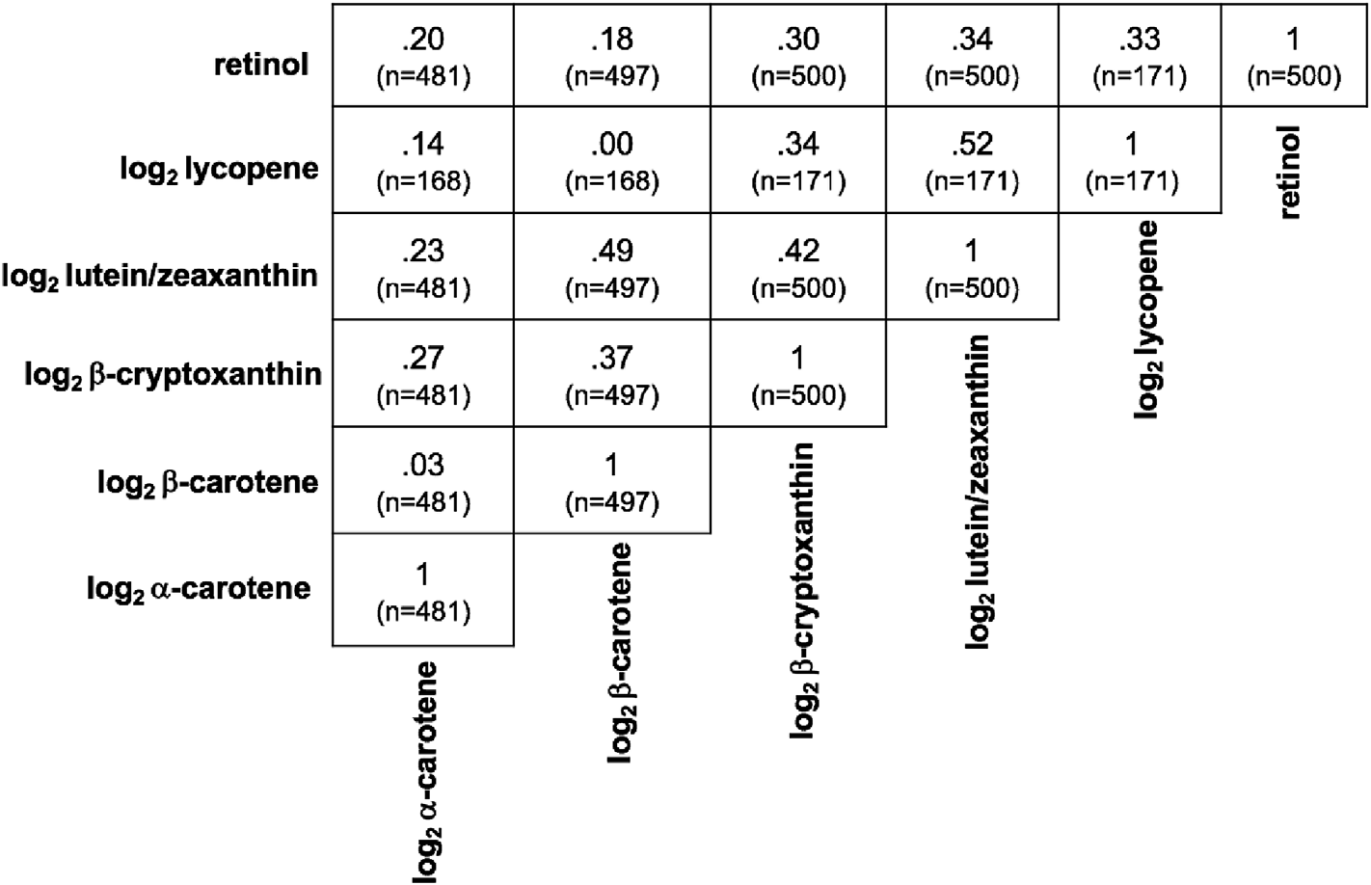
**Correlations between plasma carotenoids in 6**–**8 year old children of rural Nepal**. Correlation coefficients were generated using all complete pairwise data. All of the correlations were statistically significant (*p* < 0.0001) except log_2_ β-carotene with log_2_ α-carotene (*p* = 0.56), log_2_ β-carotene with log_2_ lycopene (*p* = 0.96) and log_2_ α-carotene with log_2_ lycopene (*p* = 0.0786).

A correlation matrix for measured carotenoids is provided for all available pairwise data in Fig. 1, with coefficients ranging from 0.03 (*p* = 0.56) to 0.52 (*p* < 0.0001). Strongest correlations were observed between lutein/zeaxanthin and lycopene (*r* = 0.52, *p* < 0.0001) and β-carotene (*r* = 0.49, *p* < 0.0001). Significant and comparable correlations also existed between β-cryptoxanthin and both lutein/zeaxanthin (r = 0.42, *p* < 0.0001) and β-carotene (*r* = 0.37, *p* < 0.0001). The prevalence of vitamin A deficiency (plasma retinol < 0.7 μmol/L) was 8.8 %, as reported [23].

Of the 982 detected proteins, 4 were associated with plasma β-carotene, 11 with lutein/zeaxanthin, and 51 with β-cryptoxanthin, meeting a FDR threshold of 10% (*q* < 0.10). No proteins met this criterion of association for plasma α-carotene or lycopene.

Among the 4 proteins associated with plasma log_2_ β-carotene, only apoliporotein-A1 (APOA1) was positively associated, while orosomucoid 1 (ORM1), pyruvate kinase muscle (PKM) and TNFAIP3 interacting protein 1 (TNIP1) were negatively associated with this carotenoid (Table 2).

**Table 2:** Plasma proteins associated with plasma log_2_ β-carotene in 6–8 year old children of rural Nepal (n = 497).^a^

Gene Name	Gene Symbol	*n*^b^	*r*	*R*^2^	*p*	*q*	*b*_*1*_^c^	gi Accession Number^d^
Orosomucoid 1	*ORM1*	497	−0.7	0.49	6.07 × 10^−5^	2.60 × 10^−2^	−33.0	167857790
Apolipoprotein A-I	*APOA1*	497	0.7	0.49	6.28 × 10^−5^	2.60 × 10^−2^	94.1	4557321
Pyruvate kinase, muscle	*PKM*	55	−0.65	0.42	1.78 × 10^−4^	4.94 × 10^−2^	−67.7	33286422
TNFAIP3 interacting protein 1	*TNIP1*	385	−0.69	0.48	1.79 × 10^−4^	4.94 × 10^−2^	−35.6	116256481

a Four proteins quantified by mass spectrometry and estimated by linear mixed effects (LME) modelling in >10% of the samples (50 < n ≤ 497) that are correlated with plasma log_2_ β-carotene, subjected to a false discovery rate (FDR) cutoff of 10% (*q* < 0.10), and listed in increasing order of *q*, defined as candidate protein biomarkers for a plasma β-carotene proteome.

b n represents the number of child plasma samples in which a protein was detected and quantified by iTRAQ MS.

c *b*_*1*_ represents the percent change in plasma β-carotene, (in μmol/L) per 2-fold (100%) increase in protein relative abundance.

d GenInfo Identifier sequence number, as assigned to all nucleotide and protein sequences by the National Center for Biotechnology Information at the National Library of Medicine, National Institutes of Health, Bethesda, MD, USA.

Seven proteins, apoliprotein-C3 (APOC3), heat shock 70 kDa protein 1A (HSPA1A), collagen type V alpha 1 (COL5A1), carnosine dipeptidase 1(CNDP1), interferon-related developmental regulator (IFRD2), APOA1, and proteoglycan 4 (PRG4) were positively associated with plasma log_2_ lutein/zeaxanthin. Four, including inter-alpha-trypsin inhibitor heavy chain 3 (ITIH3), minichromosome maintenance complex 2 (MCM2), TNIP1, and CD14 molecule (CD14) protein were negatively associated with log_2_ lutein/zeaxanthin (Table 3).

**Table 3:** Plasma proteins associated with plasma log_2_ lutein/zeaxanthin in 6–8 year old children of rural Nepal (n = 500).^a^

Gene Name	Gene Symbol	*n*^b^	*r*	*R*^2^	*p*	*q*	*b*_*1*_^c^	gi Accession Number^d^
Apolipoprotein C-III	*APOC3*	500	0.59	0.34	9.15 × 10^−5^	5.12 × 10^−2^	25.4	4557323
Inter-alpha-trypsin inhibitor heavy chain 3	*ITIH3*	500	−0.58	0.34	1.21 × 10^−4^	5.12 × 10^−2^	−28.2	133925809
Heat shock 70 kDa protein 1A	*HSPA1A*	119	0.51	0.26	1.61 × 10^−4^	5.43 × 10^−2^	69.9	194248072
Collagen, type V, alpha 1	*COL5A1*	56	0.59	0.35	3.57 × 10^−4^	8.40 × 10^−2^	101.3	89276751
Minichromosome maintenance complex component 2	*MCM2*	119	−0.64	0.41	4.95 × 10^−4^	8.40 × 10^−2^	−31.8	33356547
TNFAIP3 interacting protein 1	*TNIP1*	388	−0.6	0.36	5.43 × 10^−4^	8.40 × 10^−2^	−22.4	116256481
CD14 molecule	*CD14*	500	−0.58	0.34	5.57 × 10^−4^	8.40 × 10^−2^	−37.7	4557417
Carnosine dipeptidase 1 (metallopeptidase M20 family)	*CNDP1*	500	0.58	0.34	6.12 × 10^−4^	8.40 × 10^−2^	16.8	21071039
Interferon-related developmental regulator 2	*IFRD2*	486	0.59	0.35	6.47 × 10^−4^	8.40 × 10^−2^	27.8	197333755
Apolipoprotein A-I	*APOA1*	500	0.58	0.34	6.48 × 10^−4^	8.40 × 10^−2^	43.3	4557321
Proteoglycan 4	*PRG4*	403	0.6	0.36	7.84 × 10^−4^	9.44 × 10^−2^	46.7	189181724

a Eleven proteins quantified by mass spectrometry and estimated by linear mixed effects (LME) modelling in >10% of the samples (50 < n ≤ 500) that are correlated with plasma log_2_ lutein/zeaxanthin, subjected to a false discovery rate (FDR) cutoff of 10% (*q* < 0.10), and listed in increasing order of *q*, defined as candidate protein biomarkers for a plasma proteome of lutein/zeaxanthin.

b n represents the number of child plasma samples in which a protein was detected and quantified by iTRAQ MS.

c *b*_*1*_ represents the percent change in plasma lutein/zeaxanthin, (in μmol/L) per 2-fold (100%) increase in protein relative abundance.

d GenInfo Identifier sequence number, as assigned to all nucleotide and protein sequences by the National Center for Biotechnology Information at the National Library of Medicine, National Institutes of Health, Bethesda, MD, USA.

Among the 51 proteins associated with log_2_ β-cryptoxanthin, 31 were positive correlates (Table 4), of which 10 met a more stringent FDR of <1%, including APOA1, IFRD2, CNDP1, APOC3, phospholipid transfer protein (PLTP), PILR alpha associated neural protein (PIANP), anthrax toxin receptor 2 (ANTXR2), selenoprotein P plasma 1 (SEPP1), clusterin (CLU), and insulin-like growth factor binding protein, acid labile subunit (IGFALS). Other positively correlated plasma proteins included retinol binding protein 4 (RBP4), paraoxonase 1 (PON1), prenylcysteine oxidase 1 (PCYOX1), lymphatic vessel endothelial hyaluronan receptor 1 (LYVE1), and insulin-like growth factor (IGFBP3).

**Table 4:** Plasma proteins positively associated with plasma log_2_ β-cryptoxanthin in 6–8 year old children of rural Nepal (n = 500).^a^

Gene Name	Gene Symbol	*n*^b^	*r*	*R*^2^	*p*	*q*	*b*_*1*_^c^	gi Accession Number^d^
Apolipoprotein A-I	*APOA1*	500	0.52	0.27	5.27 × 10^−9^	7.95 × 10^−6^	193.1	4557321
Interferon-related developmental regulator 2	*IFRD2*	486	0.51	0.26	1.33 × 10^−7^	1.00 × 10^−4^	99.4	197333755
Carnosine dipeptidase 1 (metallopeptidase M20 family)	*CNDP1*	500	0.50	0.25	5.41 × 10^−6^	1.63 × 10^−3^	44.0	21071039
Apolipoprotein C-III	*APOC3*	500	0.50	0.25	8.47 × 10^−6^	2.13 × 10^−3^	58.1	4557323
Phospholipid transfer protein	*PLTP*	472	0.49	0.24	1.09 × 10^−5^	2.36 × 10^−3^	133.7	5453914
PILR alpha associated neural protein	*PIANP*	360	0.49	0.24	1.85 × 10^−5^	3.49 × 10^−3^	191.8	24308547
Anthrax toxin receptor 2	*ANTXR2*	367	0.53	0.28	2.11 × 10^−5^	3.53 × 10^−3^	95.5	50513243
Selenoprotein P, plasma, 1	*SEPP1*	500	0.50	0.25	2.69 × 10^−5^	4.06 × 10^−3^	96.7	62530391
Clusterin	*CLU*	493	0.50	0.25	5.73 × 10^−5^	7.20 × 10^−3^	300.1	42740907
Insulin-like growth factor binding protein, acid labile subunit	*IGFALS*	500	0.49	0.24	8.82 × 10^−5^	8.87 × 10^−3^	71.2	4826772
Protein phosphatase, Mg2+/Mn2+ dependent, 1 M	*PPM1M*	98	0.57	0.33	1.24 × 10^−4^	1.17 × 10^−2^	14.2	171460934
Apolipoprotein A-II	*APOA2*	500	0.49	0.24	1.52 × 10^−4^	1.35 × 10^−2^	90.4	4502149
Paraoxonase 1	*PON1*	500	0.49	0.24	2.03 × 10^−4^	1.51 × 10^−2^	61.5	19923106
Lymphatic vessel endothelial hyaluronan receptor 1	*LYVE1*	479	0.5	0.25	2.10 × 10^−4^	1.51 × 10^−2^	64.4	40549451
Retinol binding protein 4, plasma	*RBP4*	500	0.49	0.24	2.80 × 10^−4^	1.69 × 10^−2^	75.6	55743122
Eukaryotic translation initiation factor 2D	*EIF2D*	256	0.52	0.27	3.31 × 10^−4^	1.82 × 10^−2^	82.1	56699485
Apolipoprotein D	*APOD*	500	0.49	0.24	3.38 × 10^−4^	1.82 × 10^−2^	71.5	4502163
Kelch-like family member 34	*KLHL34*	403	0.51	0.26	3.90 × 10^−4^	2.03 × 10^−2^	90.3	23397572
Kruppel-like factor 17	*KLF17*	284	0.51	0.26	4.69 × 10^−4^	2.36 × 10^−2^	52.5	104294874
Prenylcysteine oxidase 1	*PCYOX1*	493	0.48	0.23	5.48 × 10^−4^	2.43 × 10^−2^	68.4	166795301
Gelsolin	*GSN*	493	0.49	0.24	5.93 × 10^−4^	2.47 × 10^−2^	92.5	4504165
Thrombospondin 4	*THBS4*	451	0.5	0.25	6.08 × 10^−4^	2.47 × 10^−2^	49.5	31543806
Apolipoprotein C-I	*APOC1*	500	0.49	0.24	6.63 × 10^−4^	2.50 × 10^−2^	27.8	4502157
Dipeptidyl-peptidase 4	*DPP4*	416	0.49	0.24	7.05 × 10^−4^	2.60 × 10^−2^	94.0	18765694
Interleukin 1 receptor accessory protein	*IL1RAP*	321	0.48	0.23	8.68 × 10^−4^	3.05 × 10^−2^	93.5	19882209
Peptidase inhibitor 16	*PI16*	500	0.49	0.24	1.01 × 10^−3^	3.46 × 10^−2^	64.3	70780384
Lumican	*LUM*	500	0.49	0.24	1.01 × 10^−3^	3.59 × 10^−2^	86.2	4505047
Insulin-like growth factor binding protein 3	*IGFBP3*	500	0.49	0.24	1.87 × 10^−3^	5.46 × 10^−2^	49.3	62243068
Cartilage oligomeric matrix protein	*COMP*	500	0.49	0.24	1.88 × 10^−3^	5.46 × 10^−2^	52.3	40217843
Glycosylphosphatidylinositol specific phospholipase D1	*GPLD1*	500	0.49	0.24	2.93 × 10^−3^	7.76 × 10^−2^	72.8	29171717
Nucleolar protein 12	*NOL12*	228	0.52	0.27	3.39 × 10^−3^	8.53 × 10^−2^	239.9	13236553

a Thirty-one proteins quantified by mass spectrometry and estimated by linear mixed effects (LME) modelling in >10% of the samples that were positively correlated with plasma log_2_ β-cryptoxanthin (*p* < 0.01, *q* < 0.10), listed in increasing order of *q*, defined as positively associated protein biomarkers of a plasma β-cryptoxanthin.

b n represents the number of child plasma samples in which a protein was detected and quantified by iTRAQ tandem MS (excludes subsequent imputations required for multivariable LME models).

c *b*_*1*_ represents the percent change in plasma β-cryptoxanthin (in μmol/L) per 2-fold (100%) increase in protein relative abundance.

d GenInfo Identifier sequence number, as assigned to all nucleotide and protein sequences by the National Center for Biotechnology Information at the National Library of Medicine, National Institutes of Health, Bethesda, MD, USA.

Among 20 plasma proteins negatively associated with log_2_ β-cryptoxanthin (Table 5), 5 met an FDR < 1%, including ORM1, TNIP1, mannosidase alpha class 1A (MAN1A1), haptoglobin (HP), and alpha-1-B glycoprotein (A1BG). Other negatively associated proteins included orosomucoid 2 (ORM2), complement factor B (CFB), complement 9 (C9), haptoglobin-related precursor (HPR), serine peptidase clade A, member 3 (SERPINA3), and lipopolysaccharide binding protein (LBP).

**Table 5:** Plasma proteins negatively associated with plasma log_2_ β-cryptoxanthin in 6–8 year old children of rural Nepal (n = 500).^a^

Gene Name	HUGO Gene Symbol	*n*^b^	*R*	*R*^2^	*p*	*q*	*b*_*1*_^c^	Accession Number^d^
Orosomucoid 1	*ORM1*	500	−0.50	0.25	1.60 × 10^−6^	8.04 × 10^−4^	−41.5	167857790
TNFAIP3 interacting protein 1	*TNIP1*	388	−0.50	0.25	2.18 × 10^−6^	8.22 × 10^−4^	−46.3	116256481
Mannosidase, alpha, class 1A, member 1	*MAN1A1*	500	−0.50	0.25	4.17 × 10^−5^	5.71 × 10^−3^	−66.0	24497519
Haptoglobin	*HP*	354	−0.53	0.28	6.94 × 10^−5^	8.06 × 10^−3^	−13.7	4826762
Alpha-1-B glycoprotein	*A1BG*	500	−0.50	0.25	8.04 × 10^−5^	8.66 × 10^−3^	−67.1	21071030
Haptoglobin-related protein	*HPR*	431	−0.52	0.27	1.83 × 10^−4^	1.51 × 10^−2^	−22.0	45580723
Leucine-rich alpha-2-glycoprotein 1	*LRG1*	500	−0.49	0.24	2.76 × 10^−4^	1.69 × 10^−2^	−37.0	16418467
Serpin peptidase inhibitor, clade A (alpha-1 antiproteinase, antitrypsin), member 3	*SERPINA3*	500	−0.49	0.24	5.42 × 10^−4^	2.43 × 10^−2^	−49.1	50659080
Orosomucoid 2	*ORM2*	500	−0.49	0.24	6.37 × 10^−4^	2.43 × 10^−2^	−41.3	4505529
Leucine rich repeat containing 47	*LRRC47*	70	−0.51	0.26	7.35 × 10^−4^	2.64 × 10^−2^	−75.0	24308207
Complement factor B	*CFB*	500	−0.49	0.24	1.08 × 10^−3^	3.59 × 10^−2^	−46.1	67782358
Beta-2-microglobulin	*B2M*	493	−0.49	0.24	1.28 × 10^−3^	4.10 × 10^−2^	−32.7	4757826
Ecotropic viral integration site 5	*EVI5*	271	−0.54	0.29	1.58 × 10^−3^	4.95 × 10^−2^	−34.6	68299759
Ubiquitin-conjugating enzyme E2L 3	*UBE2L3*	144	−0.33	0.11	1.68 × 10^−3^	5.18 × 10^−2^	−44.2	4507789
Component of oligomeric golgi complex 3	*COG3*	215	−0.31	0.09	1.76 × 10^−3^	5.30 × 10^−2^	−35.8	13899251
Lipopolysaccharide binding protein	*LBP*	500	−0.49	0.24	2.09 × 10^−3^	5.73 × 10^−2^	−28.4	31652249
Complement component 9	*C9*	500	−0.49	0.24	2.32 × 10^−3^	6.26 × 10^−2^	−38.6	4502511
Inter-alpha-trypsin inhibitor heavy chain family, member 4	*ITIH4*	500	−0.49	0.24	3.12 × 10^−3^	8.03 × 10^−2^	−58.1	31542984
Mitogen-activated protein kinase kinase kinase 14	*MAP3K14*	307	−0.50	0.25	3.14 × 10^−3^	8.03 × 10^−2^	−28.4	115298645
Lysozyme	*LYZ*	493	−0.49	0.24	3.74 × 10^−3^	9.25 × 10^−2^	−34.6	4557894

a Twenty proteins quantified by mass spectrometry and estimated by linear mixed effects (LME) modelling in >10% of the samples that were negatively correlated with plasma log_2_ β-cryptoxanthin (*p* < 0.01, *q* < 0.10), listed in increasing order of *q*, defined as negatively associated protein biomarkers of a plasma β-cryptoxanthin.

b n represents the number of child plasma samples in which a protein was detected and quantified by iTRAQ MS (excludes subsequent imputations required for multivariable LME models).

c *b*_*1*_ represents the percent change in plasma β-cryptoxanthin (in μmol/L) per 2-fold (100%) increase in protein relative abundance.

d GenInfo Identifier sequence number, as assigned to all nucleotide and protein sequences by the National Center for Biotechnology Information at the National Library of Medicine, National Institutes of Health, Bethesda, MD, USA.

**Fig. 2:**
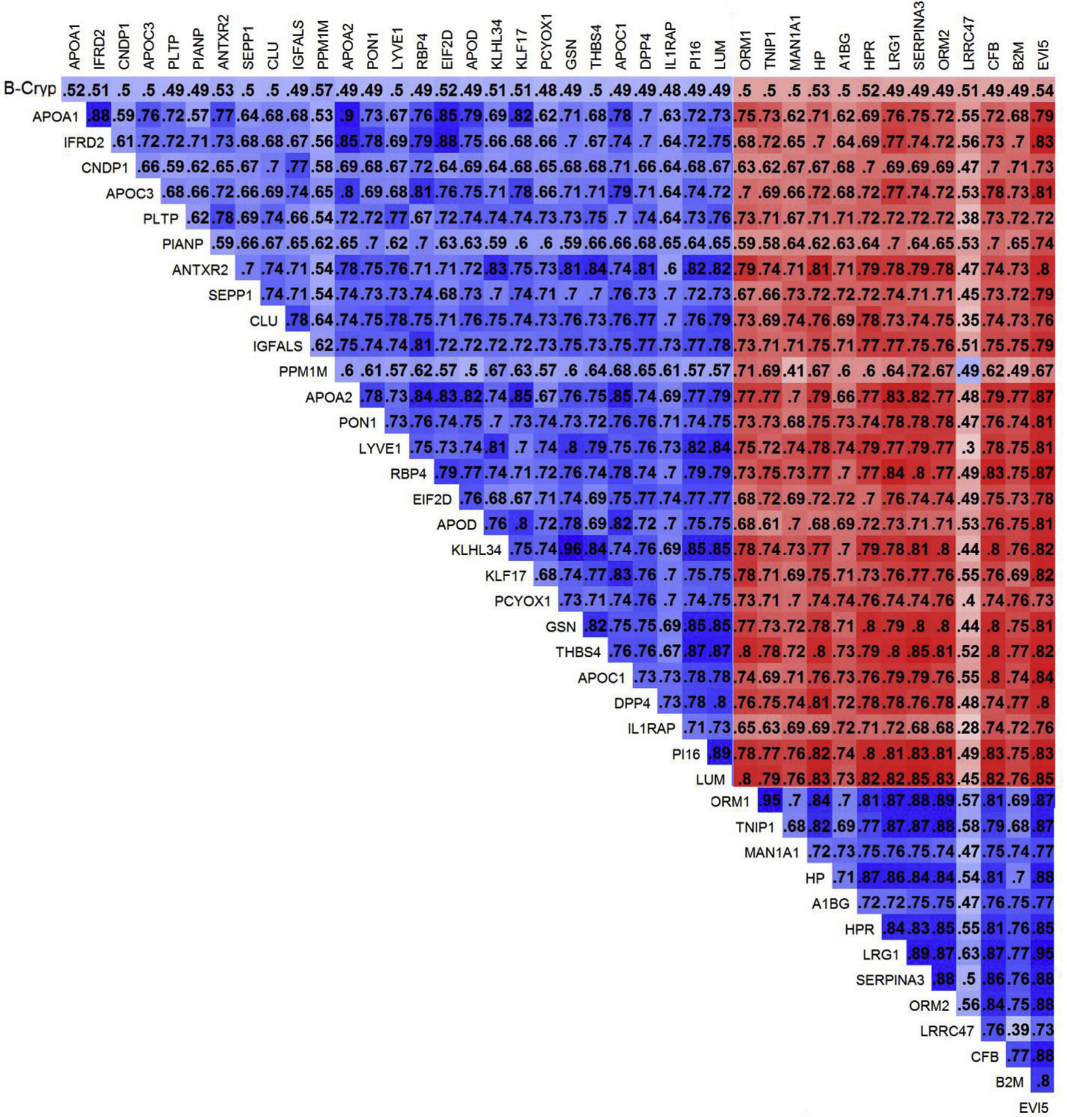
Matrix of correlation coefficients (*r*) for pairs of LME-based estimates of relative abundance estimates and plasma log_2_ β-cryptoxanthin concentration for proteins associated with β-cryptoxanthin, restricted to associations with *q* < 0.05 (n = 40), in 6–8 year old children of rural Nepal (n = 500). Blue color depicts proteins that share a positive correlation and red color a negative correlation with each other. Darker colors represent stronger association. Correlation coefficients (*r*) are presented *r* x 10^2^ to improve visualization given in each cell. APOA2, Apolipoprotein A-II; APOC1, Apolipoprotein C-I; APOD, Apolipoprotein D; B2M, β-2-microglobulin; DPP4, Dipeptidyl-peptidase 4; EIF2D, Eukaryotic translation initiation factor 2D; EVI5, ecotropic viral integration site 5; GSN, gelsolin; IL1RAP, Interleukin 1 receptor accessory protein; KLHL34, Kelch-like family member 34; KLF17, Kruppel-like factor 17; LRG1, leucine-rich α-2-glycoprotein 1; LUM, lumican; PI16, Peptidase inhibitor 16; THBS4, Thrombospondin 4.

We examined the extent of correlation across proteins associated with log_2_ β-cryptoxanthin, comprising the largest plasma carotenome, restricted to associations with FDR <5% (Fig. 2). Within each of the pairs of proteins of the β-crytoxanthin proteome, the correlation coefficients (*r*) ranged from 0.28 to 0.96. We demonstrated that proteins positively and negatively associated with β-cryptoxanthin were also consistently correlated with each other in the expected directions given their associations with β-cryptoxanthin, with the exception of protein phosphatase, Mg^2+/^Mn^2+^ dependent, 1 M (PPM1M) and leucine rich repeat containing 47 (LRRC47), which were also more weakly correlated with other proteins than most.

## 4 Discussion

Provitamin A carotenoids play important roles as dietary precursors of vitamin A that may take on particular significance in impoverished regions, such as in rural Southern Asia, where vitamin A deficiency (VAD) is endemic among young children, adolescents and women of reproductive age [11]. Carotenoids also may have important antioxidant [13], immunological [14] or metabolic [39] functions and thus serve as indicators of general population health [40]. However, given their infrequent assessment, and strengthening evidence supporting the use of plasma proteomics for assessing population status with respect to other micronutrients [[23], [24], [25], [26]], inflammation [27], cognition [28] and growth [29] in this setting, we have revealed in this study protein biomarkers associated with circulating log_2_-normalized concentrations of six common dietary carotenoids which were plausible in their direction and strength of association.

We observed four proteins associated with β-carotene, eleven with lutein/zeaxanthin, and fifty-one with β-cryptoxanthin, all with a probability of false discovery below ten percent. APOA1, a major component of high density lipoprotein (HDL) in plasma [41], was positively associated with each of the three carotenoids, possibly reflecting shared lipoprotein transport or, co-existing antioxidant, anti-inflammatory and other metabolic functions [42]. On the other hand, TNIP1, an inhibitor of the pro-inflammatory transcription factor, NF-kB [43,44] was negatively correlated with all three carotenoids. We had also shown relative abundance of TNIP1 to be positively associated with the acute phase reactant, alpha-1-acid glycoprotein (AGP), or orosomucoid, in this population [27], explained by a negative feedback loop whereby TNIP1 is upregulated by inflammation in order to maintain immune homeostasis [44]. TNIP1 also functions as a retinoic acid receptor corepresor in the presence of its ligand [45].

Nearly all proteins negatively associated across proteomes of β-carotene, lutein/zeaxanthin, and β-cryptoxanthin were previously found to be positively correlated with inflammation markers AGP and C-reactive protein (CRP) [27]. Among these proteins, complement factor B (CFB) and complement 9 (C9) are involved in regulation of complement activation [46]; haptoglobin (HP) and haptoglobin-related precursor (HPR) are responsible for scavenging of heme iron from plasma in response to inflammation and oxidative stress in red blood cells [47,48], and these proteins were negatively associated with β-cryptoxanthin. Inflammatory proteins such as AGP isoforms of orosomucoid (ORM1) -inversely associated with β-carotene- and (ORM2) [49]. Serine peptidase inhibitors, serine peptidase clade A, member 3 (SERPINA3), also known as alpha-1-antichymotrypsin, which increases in the blood during the inflammatory response [50,51], and inter-alpha-trypsin inhibitor heavy chain H4 isoform (ITIH4) as a type II acute-phase protein involved in the inflammatory response to trauma [52], were all negatively associated with plasma β-cryptoxanthin.

Somewhat surprisingly, the proteome for β-carotene, to our knowledge the most metabolically active carotenoid in human tissue and a specific vitamin A precursor, was quite limited in size (n = 4) and overlapped with that of β-cryptoxanthin, with the exception of PKM. Despite a modest, albeit universally detectable, concentration of plasma β-cryptoxanthin in the bloodstream, its proteome was far more extensive than β-carotene's. Variation in carotenoid hydrophobicity [53] may offer one explanation for this difference. Being less hydrophobic than β-carotene, β-cryptoxanthin is more likely located on the lipoprotein surface than in the core, where β-carotene is transported, thus allowing more extensive interactions with circulating proteins than possible with β-carotene.Secondly, as β-cryptoxanthin is known to be carried by HDL [54], it is notable that nearly half of the proteins found to be positively (ANTXR2, APOA1, APOA2, APOC1, APOC3, APOD, CLU, GPLD1, GSN, IGFALS, LUM, PCYOX1, PLTP, PON1 and RBP4) and negatively (CFB, C9, HP, ITIH4, LBP, ORM1, ORM2 and SERPINA3) associated with β-cryptoxanthin are known constituents of the HDL complex in human circulation [55].

Carotenoids exert their biological activity as antioxidants due to their extended conjugated carbon-carbon bonds [13]. The protective roles of carotenoids have been explored in blood plasma, where β-carotene, lutein, and zeaxanthin inhibited lipid peroxidation and hemoglobin oxidation but surprisingly lycopene and β-cryptoxanthin did not [56]. While an antioxidant function of β-cryptoxanthin has not been demonstrated in *in vivo* studies, we found it to be positively correlated with SEPP1, the major plasma carrier for selenium [57], an essential trace element that displays antioxidant activity by serving as an essential cofactor of glutathione peroxidase [58]. Plasma β-cryptoxanthin was also positively associated with PON1, an antioxidant/anti-inflammatory protein mostly synthesized by the liver and primarily associated with serum HDL [59]. To our knowledge, this is the first study demonstrating strong associations between antioxidant/anti-inflammatory PON1 and SEPP1 with plasma β-cryptoxanthin in a human population.

Plasma β-cryptoxanthin was positively correlated with relative abundance of IGFALS, IGFBP3, CNDP1, and cartilage oligomeric matrix protein (COMP), proteins that we have previously reported to be positively associated with child height and arm muscle mass in this population of school-aged Nepalese children [29], suggesting that β-cryptoxanthin nutriture, as reflected in plasma, is associated with general nutritional status, although mechanisms explaining this relationship remain unknown.

Lutein and zeaxanthin, measured together, were associated with an intermediate proteome of 11 proteins. While present in plasma, lutein and zeaxanthin are concentrated in the macula, the central region of the retina [60]. These macular carotenoids protect the retina from light-induced damage via filtering blue light [61]. Both lutein and zeaxanthin are effective antioxidants like other major carotenoids found in human plasma [62]. Lutein has been shown to protect against inflammation, by reducing the production of pro-inflammatory factors observed in retinal injury [63]. There was a positive correlation between plasma lutein/zeaxanthin and proteoglycan 4 (PRG4), a glycoprotein recently identified at the ocular surface where it functions as a lubricant [64] and its loss results in inflammation [65].

In summary, a plasma proteomics approach has revealed an extensive proteome that covaries with relative abundance of β-cryptoxanthin, despite its low circulating concentration in a generally undernourished rural population of Nepalese school-aged children. The number and diversity of plasma proteins associated with β-cryptoxanthin suggests involvement in vitamin A metabolism, lipid transport and immunoregulation. Moreover, for the first time, we speculate an *in vivo* antioxidant function of β-cryptoxanthin. Our findings suggest that plasma proteins could be measured in populations as surrogates for carotenoid intake or status, and help reveal protein:carotenoid functional relationships. More work is merited in this line of study to verify our findings and probe the implications of these novel findings for carotenoid assessment, metabolism and function and health.
