# Supplementary material for: Plasma proteins associated with circulating carotenoids in Nepalese school-aged children
Source: Arch Biochem Biophys. 2018 May 15;646:153–60. doi: 10.1016/j.abb.2018.03.025 (PMC5937903; doi:10.1016/j.abb.2018.03.025)
Supplement: Online data [file mmc2.docx]

**Plasma proteins associated with circulating carotenoids in Nepalese school-aged children**

Abdulkerim Eroglu^1,2^, Kerry J. Schulze^1,2^, James Yager^1,3^, Robert N. Cole^4,5^, Parul Christian^1,2^, Bareng A.S. Nonyane^1,2^, Sun Eun Lee^1,2^, Lee Shu Fune Wu^1,2^, Subarna Khatry^1,2^, John Groopman^1,3^, Keith P. West, Jr.^1,2^

Author affiliations: ^1^Johns Hopkins Bloomberg School of Public Health, ^2^Center for Human Nutrition, Department of International Health, ^3^Department of Environmental Health and Engineering, ^4^Johns Hopkins School of Medicine, Mass Spectrometry and Proteomics Facility, ^5^Department of Biological Chemistry Baltimore, Maryland USA

* Corresponding author: Abdulkerim Eroglu, Ph.D.; Johns Hopkins Bloomberg School of Public Health; 615 N. Wolfe St., Room W2607; Baltimore, Maryland 21205 USA; Tel: 410-614-5098; Fax: 410-955-0196; e-mail: aeroglu1@jhu.edu

List of authors’ last names: Eroglu, Schulze, Yager, Cole, Christian, Nonyane, Lee, Wu, Khatry, Groopman, West

Number of figures: 2

Number of tables: 5

Running title: Plasma proteins associated with circulating carotenoids in Nepalese school-aged children

**Abstract**

Carotenoids are naturally occurring pigments that function as vitamin A precursors, antioxidants, anti-inflammatory agents or biomarkers of recent vegetable and fruit intake, and are thus important for population health and nutritional assessment. An assay approach that measures proteins could be more technologically feasible than chromatography, thus enabling more frequent carotenoid status assessment. We explored associations between proteomic biomarkers and concentrations of 6 common dietary carotenoids (α-carotene, β-carotene, lutein/zeaxanthin, β-cryptoxanthin, and lycopene) in plasma from 500 6-8 year old Nepalese children. Samples were depleted of 6 high-abundance proteins. Plasma proteins were quantified using tandem mass spectrometry and expressed as relative abundance. Linear mixed effects models were used to determine the carotenoid:protein associations, accepting a false discovery rate of *q* < 0.10. We quantified 982 plasma proteins in > 10% of all child samples. Among these, relative abundance of 4 were associated with β-carotene, 11 with lutein/zeaxanthin and 51 with β-cryptoxanthin. Carotenoid-associated proteins are notably involved in lipid and vitamin A transport, antioxidant function and anti-inflammatory processes. No protein biomarkers met criteria for association with α-carotene or lycopene. Plasma proteomics may offer an approach to assess functional biomarkers of carotenoid status, intake and biological function for public health application.

Original maternal micronutrient trial from which data were derived as a follow-up activity was registered at ClinicalTrials.gov: NCT00115271.

**Keywords:** proteomics, plasma proteins, β-cryptoxanthin, antioxidants, carotenoids, Nepal

**Introduction**

Carotenoids are pigments occurring naturally in fruits and vegetables [1]. They are compounds synthesized from eight isoprenoid units, and more than 700 are found in nature [2-6]. Structurally, hydrocarbon carotenoids are referred to as carotenes and oxygenated carotenoids are termed xanthophylls. Among them, α-carotene, β-carotene, β-cryptoxanthin, lycopene, lutein, and zeaxanthin are the major dietary carotenoids found in human plasma [7]. While α-carotene, β-carotene and β-cryptoxanthin are provitamin A carotenoids, meaning they can be metabolized to retinol, lycopene, lutein and zeaxanthin cannot be converted to vitamin A [8]. Provitamin A carotenoids may be particularly important dietary sources for maintaining vitamin A status in impoverished regions, such as in rural Southern Asia [9], where vitamin A deficiency (VAD) has been shown to affect 20-35% of young children, school-aged adolescents and women of reproductive age [10-12] and is widely associated with low intakes of carotenoid-rich foods such as dark green leaves, yellow and orange vegetables and fruits and egg [12]. Carotenoids also appear to have *in vivo* antioxidant [13] and immunoregulatory [14] properties that are thought to give rise to frequent associations between their dietary intake or circulating concentrations and reduced risks of cardiovascular disease [15], various cancers [16] and macular degeneration [17,18]. Thus, plasma carotenoid concentrations may comprise a class of *in vivo* biomarkers that both reflect a diverse and nutritious diet [19-21] and nutritional, antioxidant, anti-inflammatory health of populations.

However, as molecules largely detected by chromatographic methods [22], carotenoids represent a group of micronutrients that are rarely assessed in low-middle income countries, signaling a need to explore novel approaches for their assessment in populations. In exploring plasma proteomics as an approach to ascertain potential biomarkers of micronutrient, functional, and health status in an undernourished population of school-aged children in Nepal, we have revealed associations between clusters of circulating proteins and micronutrient status (vitamins A, E, D and K, copper and selenium) [23-26], inflammation [27], cognition [28] and anthropometry [29] in an undernourished population of school-aged children in Nepal. Findings to date suggest that plasma proteomics can identify proteins predictive of nutritional and health status that are candidate biomarkers with the potential to be measured by multi-analyte approaches for protein quantification. Missing from the emerging knowledge base is evidence of proteins that reflect plasma carotenoids, which could benefit from assays more readily conducted than chromatography. The objective of this study was to explore the direction, strength and plausibility of association between plasma proteins and plasma carotenoid concentrations in a rural population of Nepalese school-aged children.

**Methods**

*Study cohort and field data collection.* We obtained plasma samples from over 3000 children 6-8 y of age living in the southern plains district of Sarlahi, Nepal, born to mothers who had previously participated in a 5-arm antenatal micronutrient supplementation trial [30]. Following stratification by original maternal supplement allocation group, 1000 samples were randomly selected (200 per original trial group) from children with multiple aliquots of plasma samples, complete data records from both the original trial and current follow-up study, and valid birth size measures for multiple biochemical assessments [31]. Of these, 500 samples were randomly chosen for proteomics analysis, maintaining original trial balance. Follow-up data were also collected on household socioeconomic characteristics, dietary frequencies and morbidity history for the previous 7 days and anthropometry (weight, height, mid-upper arm circumference), as reported earlier [24]. Weight-for-age z-score (WAZ), height-for-age z-score (HAZ) and body mass index (BMI)-for-age z-score (BMIZ) were used to characterize nutritional status [32]. Venous blood was drawn from children following an overnight fast, which was processed into plasma aliquots and stored in liquid nitrogen in a field laboratory. Frozen plasma was transported in vapor phase liquid nitrogen shippers to the micronutrient analysis laboratory at Johns Hopkins University in Baltimore, Maryland, U.S.A. and stored at -80^o^C until analysis. In both the original field trial and follow-up study, informed consents were obtained and protocols were approved by the Nepal Health Research Council in Kathmandu, Nepal, and the Institutional Review Board at Johns Hopkins Bloomberg School of Public Health in Baltimore, Maryland, USA.

*Plasma carotenoid analyses.* Plasma carotenoids including β-carotene, lutein and zeaxanthin, β-cryptoxanthin, α-carotene, and lycopene were analyzed by HPLC (Waters 2795) with a quaternary gradient pump, autosampler, photodiode array detector and Empower 2 software following the procedure of Yamini et al. [33]. The peaks of lutein and zeaxanthin could not be distinguished as they are combined. Separation of carotenoids was achieved using an Allsphere ODS-2, 5-μm, 4.6-mm column (Alltech) and a Supelguard Discovery C18 2-cm × 4.0-mm guard column (Sigma-Aldrich). The assay was calibrated using the National Institute of Standards and Technology standard reference material SRM968d.

*Plasma proteomics analysis.* Mass spectrometric and proteomics procedures developed for this study have been reported elsewhere [23, 34]. In brief, 500 plasma samples (40 μL) were immunoaffinity-depleted of six high abundance proteins (albumin, IgG, IgA, transferrin, haptoglobin and antitrypsin), which constitute 85-90% of total plasma protein content, using a Human-6 Multiple Affinity Removal System (MARS) LC column (Agilent Technologies). Protein extracts (100 μg each) were TCA/acetone precipitated, trypsin digested, labeled by isobaric mass tags (iTRAQ 8-plex reagents), and then seven samples plus one pooled sample for quality control was fractionated by strong cation exchange (SCX) chromatography and analyzed on a LTQ Orbitrap Velos mass spectrometer (Thermo Scientific). MS/MS spectra were searched against the RefSeq 40 database using Mascot (Matrix Science) through Proteome Discoverer software (version 1.3, Thermo Scientific) to quantify proteins with respect to the within-iTRAQ medians of log_2_ transformed and normalized reporter ion intensities derived from Proteome Discoverer. Data were obtained from 72 iTRAQ experiments with average 589 ± 65 proteins quantified per iTRAQ experiment. A total of 4705 proteins were detected, with 982 quantified in > 10% (n > 50) of all samples [23] and 146 proteins measured in all 500 samples, representing the plasma proteome for this study.

*Statistical analysis*

Detailed information on estimation of protein relative abundance from reporter ion intensities within each iTRAQ experiment was published elsewhere [34]. We applied linear mixed effects models (LME) to determine the association between log_2_ transformed plasma concentration of each carotenoid and the relative abundance of individual plasma proteins accounting for multiple iTRAQ experiments.

The expected values of log_2_ carotenoid concentrations for each individual protein from the LME can be expressed as

*E{N_rk_} = b_0_ + B_r_ + b_1_ P_rk_*

where *N_rk_* is the log_2_-transformed plasma concentration of each carotenoid, *k* is the index for each sample in each *r* iTRAQ experiment, and *P_rk_* is the protein relative abundance estimate. The parameter *b_0_* is the estimate of the intercept which is the overall mean concentration of each carotenoid; *B_r_* is the random deviation of experiment r from this mean; and, *b_1_* is the estimate of the slope of the nutrient:protein association. Statistical significance of a protein:nutrient association was assessed by a two-sided hypothesis test for *b_1_* = 0. For individual significant nutrient:protein correlations, a *q*-value, an adjusted *p*-value to control false discovery rate (FDR) was reported [35]. Protein:nutrient correlation (*r*) and R^2^ were calculated based on the observed plasma carotenoid concentrations and their respective best linear unbiased predictions from the LME models [36].

We present a list of all proteins with an FDR less than 10% (*q* < 0.10) in their associations with each plasma carotenoid, and their corresponding Human Genome Organization (HUGO) gene symbols [37], the number of samples with detected protein values (n), protein:nutrient correlation (*r*), the amount of variance in nutrient concentration as explained by the protein (*R^2^*), *p*-value derived from testing the fixed effects slope of carotenoid concentration on protein abundance, chance-adjusted *p*-value (*q*), the slope (*b_1_*), denoting relative (%) change in carotenoids per 2-fold (100%) increase in relative abundance of each protein and GenInfo identifier (*gi*) accession number [37] is provided in the tables.

Datasets of plasma carotenoid concentrations and protein relative abundance presented in this study are available in **Supplementary Table 1**. All analyses were conducted using open source software built under the R statistical computing environment [38].

**Results**

Nutritional status and demographic characteristics of study children (n=500) are shown in **Supplementary Table 2.** Study children were undernourished as reflected by low anthropometry Z-scores: 48.5%, 39.1%, and 16.1% of them were considered underweight, stunted, and thin (weight-for-age, height-for-age, and BMI-for-age Z-scores <-2, respectively), relative to the World Health Organization (WHO) reference population [32]. For β-carotene, 41.6 % children had plasma concentrations < 0.09 μmol/L **(Table 1),** which is considered low [33]. While the plasma xanthophyll carotenoids, β-cryptoxanthin and lutein/zeaxanthin were detected among all children, plasma carotenes, α-carotene and lycopene, were more likely to be below detection limits. Values below the detection limit were not included in the distribution of values shown in Table 1.

A correlation matrix for measured carotenoids is provided for all available pairwise data in **Figure 1,** with coefficients ranging from 0.03 (*p* = 0.56) to 0.52 (*p* < 0.0001). Strongest correlations were observed between lutein/zeaxanthin and lycopene (*r* = 0.52, *p* < 0.0001) and β-carotene (*r* = 0.49, *p* < 0.0001). Significant and comparable correlations also existed between β-cryptoxanthin and both lutein/zeaxanthin (r = 0.42, *p* < 0.0001) and β-carotene (*r* = 0.37, *p* < 0.0001).

Of the 982 detected proteins, 4 were associated with plasma β-carotene, 11 with lutein/zeaxanthin, and 51 with β-cryptoxanthin, meeting a FDR threshold of 10% (*q* < 0.10). No proteins met this criterion of association for plasma α-carotene or lycopene.

Among the 4 proteins associated with plasma log_2_ β-carotene, only apoliporotein-A1 (APOA1) was positively associated, while orosomucoid 1 (ORM1), pyruvate kinase muscle (PKM) and TNFAIP3 interacting protein 1 (TNIP1) were negatively associated with this carotenoid **(Table 2).**

Seven proteins, apoliprotein-C3 (APOC3), heat shock 70kDa protein 1A (HSPA1A), collagen type V alpha 1 (COL5A1), carnosine dipeptidase 1(CNDP1), interferon-related developmental regulator (IFRD2), APOA1, and proteoglycan 4 (PRG4) were positively associated with plasma log_2_ lutein/zeaxanthin. Four, including inter-alpha-trypsin inhibitor heavy chain 3 (ITIH3), minichromosome maintenance complex 2 (MCM2), TNIP1, and CD14 molecule (CD14) protein were negatively associated with log_2_ lutein/zeaxanthin **(Table 3).**

Among the 51 proteins associated with log_2_ β-cryptoxanthin, 31 were positive correlates **(Table 4)**, of which 10 met a more stringent FDR of < 1%, including APOA1, IFRD2, CNDP1, APOC3, phospholipid transfer protein (PLTP), PILR alpha associated neural protein (PIANP), anthrax toxin receptor 2 (ANTXR2), selenoprotein P plasma 1 (SEPP1), clusterin (CLU), and insulin-like growth factor binding protein, acid labile subunit (IGFALS)**.** Other positively correlated plasma proteins included retinol binding protein 4 (RBP4), paraoxonase 1 (PON1), prenylcysteine oxidase 1 (PCYOX1), lymphatic vessel endothelial hyaluronan receptor 1 (LYVE1), and insulin-like growth factor (IGFBP3).

Among 20 plasma proteins negatively associated with log_2_ β-cryptoxanthin **(Table 5)**, 5 met an FDR < 1%, including ORM1, TNIP1, mannosidase alpha class 1A (MAN1A1), haptoglobin (HP), and alpha-1-B glycoprotein (A1BG). Other negatively associated proteins included orosomucoid 2 (ORM2), complement factor B (CFB), complement 9 (C9), haptoglobin-related precursor (HPR), serine peptidase clade A, member 3 (SERPINA3), and lipopolysaccharide binding protein (LBP).

We examined the extent of correlation across proteins associated with log_2_ β-cryptoxanthin, comprising the largest plasma carotenome, restricted to associations with FDR < 5% (**Figure 2**). Within each of the pairs of proteins of the β-crytoxanthin proteome, the correlation coefficients (*r*) ranged from 0.28 to 0.96. We demonstrated that proteins positively and negatively associated with β-cryptoxanthin were also consistently correlated with each other in the expected directions given their associations with β-cryptoxanthin, with the exception of protein phosphatase, Mg^2+/^Mn^2+^ dependent, 1M (PPM1M) and leucine rich repeat containing 47 (LRRC47), which were also more weakly correlated with other proteins than most.

**Discussion**

Provitamin A carotenoids play important roles as dietary precursors of vitamin A that may take on particular significance in impoverished regions, such as in rural Southern Asia, where vitamin A deficiency (VAD) is endemic among young children, adolescents and women of reproductive age [11]. Carotenoids also may have important antioxidant [13], immunological [14] or metabolic [39] functions and thus serve as indicators of general population health [40]. However, given their infrequent assessment, and strengthening evidence supporting the use of plasma proteomics for assessing population status with respect to other micronutrients [23-26], inflammation [27], cognition [28] and growth [29] in this setting, we have revealed in this study protein biomarkers associated with circulating log_2_-normalized concentrations of six common dietary carotenoids which were plausible in their direction and strength of association.

We observed four proteins associated with β-carotene, eleven with lutein/zeaxanthin, and fifty-one with β-cryptoxanthin, all with a probability of false discovery below ten percent. APOA1, a major component of high density lipoprotein (HDL) in plasma [41], was positively associated with each of the three carotenoids, possibly reflecting shared lipoprotein transport or, co-existing antioxidant, anti-inflammatory and other metabolic functions [42]. On the other hand, TNIP1, an inhibitor of the pro-inflammatory transcription factor, NF-kB [43, 44] was negatively correlated with all three carotenoids. We had also shown relative abundance of TNIP1 to be positively associated with the acute phase reactant, alpha-1-acid glycoprotein (AGP), or orosomucoid, in this population [27], explained by a negative feedback loop whereby TNIP1 is upregulated by inflammation in order to maintain immune homeostasis [44].

Nearly all proteins negatively associated across proteomes of β-carotene, lutein/zeaxanthin, and β-cryptoxanthin were previously found to be positively correlated with inflammation markers AGP and C-reactive protein (CRP) [27]. Among these proteins, complement factor B (CFB) and complement 9 (C9) are involved in regulation of complement activation [45]; haptoglobin (HP) and haptoglobin-related precursor (HPR) are responsible for scavenging of heme iron from plasma in response to inflammation and oxidative stress in red blood cells [46, 47], and these proteins were negatively associated with β-cryptoxanthin. Inflammatory proteins such as AGP isoforms of orosomucoid (ORM1) -inversely associated with β-carotene- and (ORM2) [48]. Serine peptidase inhibitors, serine peptidase clade A, member 3 (SERPINA3), also known as alpha-1-antichymotrypsin, which increases in the blood during the inflammatory response [49, 50], and inter-alpha-trypsin inhibitor heavy chain H4 isoform (ITIH4) as a type II acute-phase protein involved in the inflammatory response to trauma [51], were all negatively associated with plasma β-cryptoxanthin.

Somewhat surprisingly, the proteome for β-carotene, to our knowledge the most metabolically active carotenoid in human tissue and a specific vitamin A precursor, was quite limited in size (n=4) and overlapped with that of β-cryptoxanthin, with the exception of PKM. Despite a modest, albeit universally detectable, concentration of plasma β-cryptoxanthin in the bloodstream, its proteome was far more extensive than β-carotene’s. Because it is less hydrophobic than β-carotene, β-cryptoxanthin is located on the surface of carrier apolipoproteins rather than in the inner core where β-carotene is transported, possibly allowing more extensive interactions with circulating proteins than β-carotene, explaining its larger proteome. Among the half-dozen lipid transport proteins with which β-cryptoxanthin was associated was the lipocalin retinol binding protein (RBP4), in addition to retinol itself.

Carotenoids exert their biological activity as antioxidants due to their extended conjugated carbon-carbon bonds [13]. The protective roles of carotenoids have been explored in blood plasma, where β-carotene, lutein, and zeaxanthin inhibited lipid peroxidation and hemoglobin oxidation but surprisingly lycopene and β-cryptoxanthin did not [52]. While an antioxidant function of β-cryptoxanthin has not been demonstrated in *in vivo* studies, we found it to be positively correlated with SEPP1, the major plasma carrier for selenium [53], an essential trace element that displays antioxidant activity by serving as an essential cofactor of glutathione peroxidase [54]. Plasma β-cryptoxanthin was also positively associated with PON1, an antioxidant/anti-inflammatory protein mostly synthesized by the liver and primarily associated with serum HDL [55]. To our knowledge, this is the first study demonstrating strong associations between antioxidant/anti-inflammatory PON1 and SEPP1 with plasma β-cryptoxanthin in a human population.

Plasma β-cryptoxanthin was positively correlated with relative abundance of IGFALS, IGFBP3, CNDP1, and cartilage oligomeric matrix protein (COMP), proteins that we have previously reported to be positively associated with child height and arm muscle mass in this population of school-aged Nepalese children [29], suggesting that β-cryptoxanthin nutriture, as reflected in plasma, is associated with general nutritional status, although mechanisms explaining this relationship remain unknown.

Lutein and zeaxanthin, measured together, demonstrated an intermediate sized proteome comprised of 11 proteins. While present in plasma, lutein and zeaxanthin are concentrated in the macula, the central region of the retina [56]. These macular carotenoids protect the retina from light-induced damage via filtering blue light [57]. Both lutein and zeaxanthin are effective antioxidants like other major carotenoids found in human plasma [58]. Lutein has been shown to protect against inflammation, by reducing the production of pro-inflammatory factors observed in retinal injury [59]. There was a positive correlation between plasma lutein/zeaxanthin and proteoglycan 4 (PRG4), a glycoprotein recently identified at the ocular surface where it functions as a lubricant [60] and its loss results in inflammation [61].

In summary, a plasma proteomics approach has revealed an extensive proteome that covaries with relative abundance of β-cryptoxanthin, despite its low circulating concentration in a generally undernourished rural population of Nepalese school-aged children. The number and diversity of plasma proteins associated with β-cryptoxanthin suggests involvement in vitamin A metabolism, lipid transport and immunoregulation. Moreover, for the first time, we speculate an *in vivo* antioxidant function of β-cryptoxanthin. Our findings suggest that plasma proteins could be measured in populations as surrogates for carotenoid intake or status, and help reveal protein:carotenoid functional relationships. More work is merited in this line of study to verify our findings and probe the implications of these novel findings for carotenoid assessment, metabolism and function and health.

**Acknowledgements**

We thank the Johns Hopkins nutriproteomics research team including Margia Arguello, Raghothama Chaerkady, Hongie Cui, Lauren R DeVine, Jaime Johnson, Robert O’Meally, Steven C LeClerq, Ashika Nanayakkara-Bind, Hee-Sool Rho, Sudeep Shrestha, and Fredrick Van Dyke for assistance with the nutriproteomics research and Nepal field activities. We thank Ingo Ruczinski for his proteomics modeling and analytic guidance and C Conover Talbot Jr. for assistance with the Human Genome Organization gene annotation.

**Footnotes**

## This study was supported by the Assessment of Micronutrient Status by Nutriproteomics Grant OPP 5241 from the Bill & Melinda Gates Foundation (Yiwu He, Senior Program Officer). The cohort study in Nepal from which plasma samples were obtained was supported by the Global Control of Micronutrient Deficiency Grant GH 614 from the Bill & Melinda Gates Foundation (Ellen Piwoz, Senior Program Officer). The original field trial in Nepal from 1999 to 2001 in which mothers of studied children were enrolled was supported by Micronutrients for Health Cooperative Agreement HRN-A-00-97-00015-00 between the Office of Health, Infectious Diseases and Nutrition, US Agency for International Development and the Center for Human Nutrition, Johns Hopkins Bloomberg School of Public Health. The Sight and Life Global Nutrition Research Institute also provided additional technical assistance.

**Table 1. Plasma carotenoid concentrations (µmol/L) of children, 6-8 years of age, Sarlahi, Nepal**

| Plasma carotenoids | n^1^ | Median (IQR)^2^ |
| --- | --- | --- |
| β-Carotene^3^ | 497 | 0.10 (0.06, 0.19) |
| Lutein/zeaxanthin | 500 | 0.34 (0.25, 0.48) |
| β-Cryptoxanthin | 500 | 0.06 (0.04, 0.12) |
| α-Carotene | 481 | 0.01 (0.01, 0.02) |
| Lycopene | 171 | 0.02 (0.01, 0.03) |

^1^ n represents the number of samples with detectable concentration of each carotenoid

^2^ Values are median (1^st^ IQR, 3^rd^ IQR).

^3^ Cutoff for low plasma β-carotene is 0.09 μmol/L, below which 207 values were observed (41.6 %).

**Table 2. Plasma proteins associated with plasma log_2_ β-carotene in 6-8 year old children of rural Nepal (n=497)^1^**

| Gene Name | Gene Symbol | *n*^2^ | *r* | *R^2^* | *p* | *q* | *b_1_ ^3^* | gi Accession  Number^4^ |
| --- | --- | --- | --- | --- | --- | --- | --- | --- |
| Orosomucoid 1 | *ORM1* | 497 | -0.7 | 0.49 | 6.07X10^-5^ | 2.60X10^-2^ | -33.0 | 167857790 |
| Apolipoprotein A-I | *APOA1* | 497 | 0.7 | 0.49 | 6.28X10^-5^ | 2.60X10^-2^ | 94.1 | 4557321 |
| Pyruvate kinase, muscle | *PKM* | 55 | -0.65 | 0.42 | 1.78X10^-4^ | 4.94X10^-2^ | -67.7 | 33286422 |
| TNFAIP3 interacting protein 1 | *TNIP1* | 385 | -0.69 | 0.48 | 1.79X10^-4^ | 4.94X10^-2^ | -35.6 | 116256481 |

^1^ Four proteins quantified by mass spectrometry and estimated by linear mixed effects (LME) modelling in > 10% of the samples (50 < n < 497) that are correlated with plasma log_2_ β-carotene, subjected to a false discovery rate (FDR) cutoff of 10% (*q* < 0.10), and listed in increasing order of *q*, defined as candidate protein biomarkers for a plasma β-carotene proteome.

^2^ n represents the number of child plasma samples in which a protein was detected and quantified by iTRAQ MS

^3^ *b_1_* represents the percent change in plasma β-carotene, (in µmol/L) per 2-fold (100%) increase in protein relative abundance.

^4^ GenInfo Identifier sequence number, as assigned to all nucleotide and protein sequences by the National Center for Biotechnology Information at the National Library of Medicine, National Institutes of Health, Bethesda, MD, USA.

**Table 3. Plasma proteins associated with plasma log_2_ lutein/zeaxanthin in 6-8 year old children of rural Nepal (n=500)^1^**

| Gene Name | Gene Symbol | *n*^2^ | *r* | *R^2^* | *p* | *q* | *b_1_* ^3^ | gi Accession  Number^4^ |
| --- | --- | --- | --- | --- | --- | --- | --- | --- |
| Apolipoprotein C-III | *APOC3* | 500 | 0.59 | 0.34 | 9.15X10^-5^ | 5.12X10^-2^ | 25.4 | 4557323 |
| Inter-alpha-trypsin inhibitor heavy chain 3 | *ITIH3* | 500 | -0.58 | 0.34 | 1.21X10^-4^ | 5.12X10^-2^ | -28.2 | 133925809 |
| Heat shock 70kDa protein 1A | *HSPA1A* | 119 | 0.51 | 0.26 | 1.61X10^-4^ | 5.43X10^-2^ | 69.9 | 194248072 |
| Collagen, type V, alpha 1 | *COL5A1* | 56 | 0.59 | 0.35 | 3.57X10^-4^ | 8.40X10^-2^ | 101.3 | 89276751 |
| Minichromosome maintenance complex component 2 | *MCM2* | 119 | -0.64 | 0.41 | 4.95X10^-4^ | 8.40X10^-2^ | -31.8 | 33356547 |
| TNFAIP3 interacting protein 1 | *TNIP1* | 388 | -0.6 | 0.36 | 5.43X10^-4^ | 8.40X10^-2^ | -22.4 | 116256481 |
| CD14 molecule | *CD14* | 500 | -0.58 | 0.34 | 5.57X10^-4^ | 8.40X10^-2^ | -37.7 | 4557417 |
| Carnosine dipeptidase 1 (metallopeptidase M20 family) | *CNDP1* | 500 | 0.58 | 0.34 | 6.12X10^-4^ | 8.40X10^-2^ | 16.8 | 21071039 |
| Interferon-related developmental regulator 2 | *IFRD2* | 486 | 0.59 | 0.35 | 6.47X10^-4^ | 8.40X10^-2^ | 27.8 | 197333755 |
| Apolipoprotein A-I | *APOA1* | 500 | 0.58 | 0.34 | 6.48X10^-4^ | 8.40X10^-2^ | 43.3 | 4557321 |
| Proteoglycan 4 | *PRG4* | 403 | 0.6 | 0.36 | 7.84X10^-4^ | 9.44X10^-2^ | 46.7 | 189181724 |

^1^ Eleven proteins quantified by mass spectrometry and estimated by linear mixed effects (LME) modelling in > 10% of the samples (50 < n < 500) that are correlated with plasma log_2_ lutein/zeaxanthin, subjected to a false discovery rate (FDR) cutoff of 10% (*q* < 0.10), and listed in increasing order of *q*, defined as candidate protein biomarkers for a plasma proteome of lutein/zeaxanthin.

^2^ n represents the number of child plasma samples in which a protein was detected and quantified by iTRAQ MS.

^3^ *b_1_* represents the percent change in plasma lutein/zeaxanthin, (in µmol/L) per 2-fold (100%) increase in protein relative abundance.

^4^ GenInfo Identifier sequence number, as assigned to all nucleotide and protein sequences by the National Center for Biotechnology Information at the National Library of Medicine, National Institutes of Health, Bethesda, MD, USA.

**Table 4. Plasma proteins positively associated with plasma log_2_ β-cryptoxanthin in 6-8 year old children of rural Nepal (n=500)^1^**

| Gene Name | Gene Symbol | *n*^2^ | *r* | *R^2^* | *p* | *q* | *b_1_* ^3^ | gi Accession  Number^4^ |
| --- | --- | --- | --- | --- | --- | --- | --- | --- |
| Apolipoprotein A-I | *APOA1* | 500 | 0.52 | 0.27 | 5.27X10^-9^ | 7.95X10^-6^ | 193.1 | 4557321 |
| Interferon-related developmental regulator 2 | *IFRD2* | 486 | 0.51 | 0.26 | 1.33X10^-7^ | 1.00X10^-4^ | 99.4 | 197333755 |
| Carnosine dipeptidase 1 (metallopeptidase M20 family) | *CNDP1* | 500 | 0.50 | 0.25 | 5.41X10^-6^ | 1.63X10^-3^ | 44.0 | 21071039 |
| Apolipoprotein C-III | *APOC3* | 500 | 0.50 | 0.25 | 8.47X10^-6^ | 2.13X10^-3^ | 58.1 | 4557323 |
| Phospholipid transfer protein | *PLTP* | 472 | 0.49 | 0.24 | 1.09X10^-5^ | 2.36X10^-3^ | 133.7 | 5453914 |
| PILR alpha associated neural protein | *PIANP* | 360 | 0.49 | 0.24 | 1.85X10^-5^ | 3.49X10^-3^ | 191.8 | 24308547 |
| Anthrax toxin receptor 2 | *ANTXR2* | 367 | 0.53 | 0.28 | 2.11X10^-5^ | 3.53X10^-3^ | 95.5 | 50513243 |
| Selenoprotein P, plasma, 1 | *SEPP1* | 500 | 0.50 | 0.25 | 2.69X10^-5^ | 4.06X10^-3^ | 96.7 | 62530391 |
| Clusterin | *CLU* | 493 | 0.50 | 0.25 | 5.73X10^-5^ | 7.20X10^-3^ | 300.1 | 42740907 |
| Insulin-like growth factor binding protein, acid labile subunit | *IGFALS* | 500 | 0.49 | 0.24 | 8.82X10^-5^ | 8.87X10^-3^ | 71.2 | 4826772 |
| Protein phosphatase, Mg2+/Mn2+ dependent, 1M | *PPM1M* | 98 | 0.57 | 0.33 | 1.24X10^-4^ | 1.17X10^-2^ | 14.2 | 171460934 |
| Apolipoprotein A-II | *APOA2* | 500 | 0.49 | 0.24 | 1.52X10^-4^ | 1.35X10^-2^ | 90.4 | 4502149 |
| Paraoxonase 1 | *PON1* | 500 | 0.49 | 0.24 | 2.03X10^-4^ | 1.51X10^-2^ | 61.5 | 19923106 |
| Lymphatic vessel endothelial hyaluronan receptor 1 | *LYVE1* | 479 | 0.5 | 0.25 | 2.10X10^-4^ | 1.51X10^-2^ | 64.4 | 40549451 |
| Retinol binding protein 4, plasma | *RBP4* | 500 | 0.49 | 0.24 | 2.80X10^-4^ | 1.69X10^-2^ | 75.6 | 55743122 |
| Eukaryotic translation initiation factor 2D | *EIF2D* | 256 | 0.52 | 0.27 | 3.31X10^-4^ | 1.82X10^-2^ | 82.1 | 56699485 |
| Apolipoprotein D | *APOD* | 500 | 0.49 | 0.24 | 3.38X10^-4^ | 1.82X10^-2^ | 71.5 | 4502163 |
| Kelch-like family member 34 | *KLHL34* | 403 | 0.51 | 0.26 | 3.90X10^-4^ | 2.03X10^-2^ | 90.3 | 23397572 |
| Kruppel-like factor 17 | *KLF17* | 284 | 0.51 | 0.26 | 4.69X10^-4^ | 2.36X10^-2^ | 52.5 | 104294874 |
| Prenylcysteine oxidase 1 | *PCYOX1* | 493 | 0.48 | 0.23 | 5.48X10^-4^ | 2.43X10^-2^ | 68.4 | 166795301 |
| Gelsolin | *GSN* | 493 | 0.49 | 0.24 | 5.93X10^-4^ | 2.47X10^-2^ | 92.5 | 4504165 |
| Thrombospondin 4 | *THBS4* | 451 | 0.5 | 0.25 | 6.08X10^-4^ | 2.47X10^-2^ | 49.5 | 31543806 |
| Apolipoprotein C-I | *APOC1* | 500 | 0.49 | 0.24 | 6.63X10^-4^ | 2.50X10^-2^ | 27.8 | 4502157 |
| Dipeptidyl-peptidase 4 | *DPP4* | 416 | 0.49 | 0.24 | 7.05X10^-4^ | 2.60X10^-2^ | 94.0 | 18765694 |
| Interleukin 1 receptor accessory protein | *IL1RAP* | 321 | 0.48 | 0.23 | 8.68X10^-4^ | 3.05X10^-2^ | 93.5 | 19882209 |
| Peptidase inhibitor 16 | *PI16* | 500 | 0.49 | 0.24 | 1.01X10^-3^ | 3.46X10^-2^ | 64.3 | 70780384 |
| Lumican | *LUM* | 500 | 0.49 | 0.24 | 1.01X10^-3^ | 3.59X10^-2^ | 86.2 | 4505047 |
| Insulin-like growth factor binding protein 3 | *IGFBP3* | 500 | 0.49 | 0.24 | 1.87X10^-3^ | 5.46X10^-2^ | 49.3 | 62243068 |
| Cartilage oligomeric matrix protein | *COMP* | 500 | 0.49 | 0.24 | 1.88X10^-3^ | 5.46X10^-2^ | 52.3 | 40217843 |
| Glycosylphosphatidylinositol specific phospholipase D1 | *GPLD1* | 500 | 0.49 | 0.24 | 2.93X10^-3^ | 7.76X10^-2^ | 72.8 | 29171717 |
| Nucleolar protein 12 | *NOL12* | 228 | 0.52 | 0.27 | 3.39X10^-3^ | 8.53X10^-2^ | 239.9 | 13236553 |

^1^ Thirty-one proteins quantified by mass spectrometry and estimated by linear mixed effects (LME) modelling in >10% of the samples that were positively correlated with plasma log_2_ β-cryptoxanthin (*p* < 0.01, *q* < 0.10), listed in increasing order of *q*, defined as positively associated protein biomarkers of a plasma β-cryptoxanthin.

^2^ n represents the number of child plasma samples in which a protein was detected and quantified by iTRAQ tandem MS (excludes subsequent imputations required for multivariable LME models).

^3^ *b_1_* represents the percent change in plasma β-cryptoxanthin (in μmol/L) per 2-fold (100%) increase in protein relative abundance.

^4^ GenInfo Identifier sequence number, as assigned to all nucleotide and protein sequences by the National Center for Biotechnology Information at the National Library of Medicine, National Institutes of Health, Bethesda, MD, USA.

**Table 5. Plasma proteins negatively associated with plasma log_2_ β-cryptoxanthin in 6-8 year old children of rural Nepal (n=500)^1^**

| Gene Name | HUGO  Gene Symbol | *n*^2^ | *R* | *R^2^* | *p* | *q* | *b_1_* ^3^ | Accession  Number^4^ |
| --- | --- | --- | --- | --- | --- | --- | --- | --- |
| Orosomucoid 1 | *ORM1* | 500 | -0.50 | 0.25 | 1.60X10^-6^ | 8.04X10^-4^ | -41.5 | 167857790 |
| TNFAIP3 interacting protein 1 | *TNIP1* | 388 | -0.50 | 0.25 | 2.18X10^-6^ | 8.22X10^-4^ | -46.3 | 116256481 |
| Mannosidase, alpha, class 1A, member 1 | *MAN1A1* | 500 | -0.50 | 0.25 | 4.17X10^-5^ | 5.71X10^-3^ | -66.0 | 24497519 |
| Haptoglobin | *HP* | 354 | -0.53 | 0.28 | 6.94X10^-5^ | 8.06X10^-3^ | -13.7 | 4826762 |
| Alpha-1-B glycoprotein | *A1BG* | 500 | -0.50 | 0.25 | 8.04X10^-5^ | 8.66X10^-3^ | -67.1 | 21071030 |
| Haptoglobin-related protein | *HPR* | 431 | -0.52 | 0.27 | 1.83X10^-4^ | 1.51X10^-2^ | -22.0 | 45580723 |
| Leucine-rich alpha-2-glycoprotein 1 | *LRG1* | 500 | -0.49 | 0.24 | 2.76X10^-4^ | 1.69X10^-2^ | -37.0 | 16418467 |
| Serpin peptidase inhibitor, clade A (alpha-1 antiproteinase, antitrypsin), member 3 | *SERPINA3* | 500 | -0.49 | 0.24 | 5.42X10^-4^ | 2.43X10^-2^ | -49.1 | 50659080 |
| Orosomucoid 2 | *ORM2* | 500 | -0.49 | 0.24 | 6.37X10^-4^ | 2.43X10^-2^ | -41.3 | 4505529 |
| Leucine rich repeat containing 47 | *LRRC47* | 70 | -0.51 | 0.26 | 7.35X10^-4^ | 2.64X10^-2^ | -75.0 | 24308207 |
| Complement factor B | *CFB* | 500 | -0.49 | 0.24 | 1.08X10^-3^ | 3.59X10^-2^ | -46.1 | 67782358 |
| Beta-2-microglobulin | *B2M* | 493 | -0.49 | 0.24 | 1.28X10^-3^ | 4.10X10^-2^ | -32.7 | 4757826 |
| Ecotropic viral integration site 5 | *EVI5* | 271 | -0.54 | 0.29 | 1.58X10^-3^ | 4.95X10^-2^ | -34.6 | 68299759 |
| Ubiquitin-conjugating enzyme E2L 3 | *UBE2L3* | 144 | -0.33 | 0.11 | 1.68X10^-3^ | 5.18X10^-2^ | -44.2 | 4507789 |
| Component of oligomeric golgi complex 3 | *COG3* | 215 | -0.31 | 0.09 | 1.76X10^-3^ | 5.30X10^-2^ | -35.8 | 13899251 |
| Lipopolysaccharide binding protein | *LBP* | 500 | -0.49 | 0.24 | 2.09X10^-3^ | 5.73X10^-2^ | -28.4 | 31652249 |
| Complement component 9 | *C9* | 500 | -0.49 | 0.24 | 2.32X10^-3^ | 6.26X10^-2^ | -38.6 | 4502511 |
| Inter-alpha-trypsin inhibitor heavy chain family, member 4 | *ITIH4* | 500 | -0.49 | 0.24 | 3.12X10^-3^ | 8.03X10^-2^ | -58.1 | 31542984 |
| Mitogen-activated protein kinase kinase kinase 14 | *MAP3K14* | 307 | -0.50 | 0.25 | 3.14X10^-3^ | 8.03X10^-2^ | -28.4 | 115298645 |
| Lysozyme | *LYZ* | 493 | -0.49 | 0.24 | 3.74X10^-3^ | 9.25X10^-2^ | -34.6 | 4557894 |

^1^ Twenty proteins quantified by mass spectrometry and estimated by linear mixed effects (LME) modelling in >10% of the samples that were negatively correlated with plasma log_2_ β-cryptoxanthin (*p* < 0.01, *q* < 0.10), listed in increasing order of *q*, defined as negatively associated protein biomarkers of a plasma β-cryptoxanthin.

^2^ n represents the number of child plasma samples in which a protein was detected and quantified by iTRAQ MS (excludes subsequent imputations required for multivariable LME models).

^3^ *b_1_* represents the percent change in plasma β-cryptoxanthin (in μmol/L) per 2-fold (100%) increase in protein relative abundance.

^4^ GenInfo Identifier sequence number, as assigned to all nucleotide and protein sequences by the National Center for Biotechnology Information at the National Library of Medicine, National Institutes of Health, Bethesda, MD, USA

**Figure 1. Correlations between plasma carotenoids in 6-8 year old children of rural Nepal**. Correlation coefficients were generated using all complete pairwise data. All of the correlations were statistically significant (*p* < 0.0001) except log_2_ β-carotene with log_2_ α-carotene (*p* = 0.56), log_2_ β-carotene with log_2_ lycopene (*p* = 0.96) and log_2_ α-carotene with log_2_ lycopene (*p* = 0.0786).

**
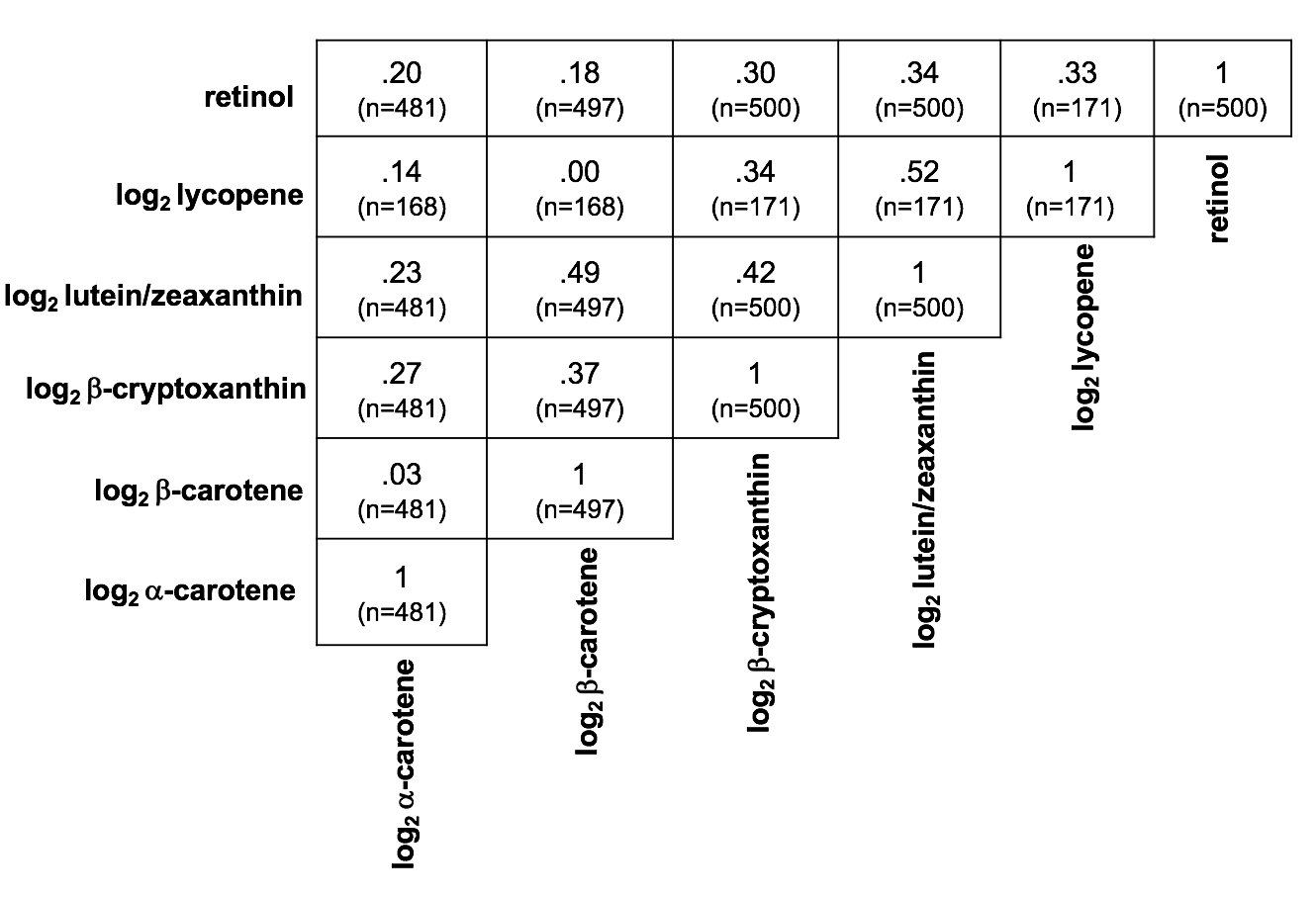
**

**Figure 2.** Matrix of correlation coefficients (*r*) for pairs of LME-based estimates of relative abundance estimates and plasma log_2_ β-cryptoxanthin concentration for proteins associated with β-cryptoxanthin, restricted to associations with *q* < 0.05 (n = 40), in 6-8 year old children of rural Nepal (n=500). Blue color depicts proteins that share a positive correlation and red color a negative correlation with each other. Darker colors represent stronger association. Correlation coefficients (*r*) are presented *r* x 10^2^ to improve visualization given in each cell. APOA2, Apolipoprotein A-II; APOC1, Apolipoprotein C-I; APOD, Apolipoprotein D; B2M, β-2-microglobulin; DPP4, Dipeptidyl-peptidase 4; EIF2D, Eukaryotic translation initiation factor 2D; EVI5, ecotropic viral integration site 5; GSN, gelsolin; IL1RAP, Interleukin 1 receptor accessory protein; KLHL34, Kelch-like family member 34; KLF17, Kruppel-like factor 17; LRG1, leucine-rich α-2-glycoprotein 1; LUM, lumican; PI16, Peptidase inhibitor 16; THBS4, Thrombospondin 4


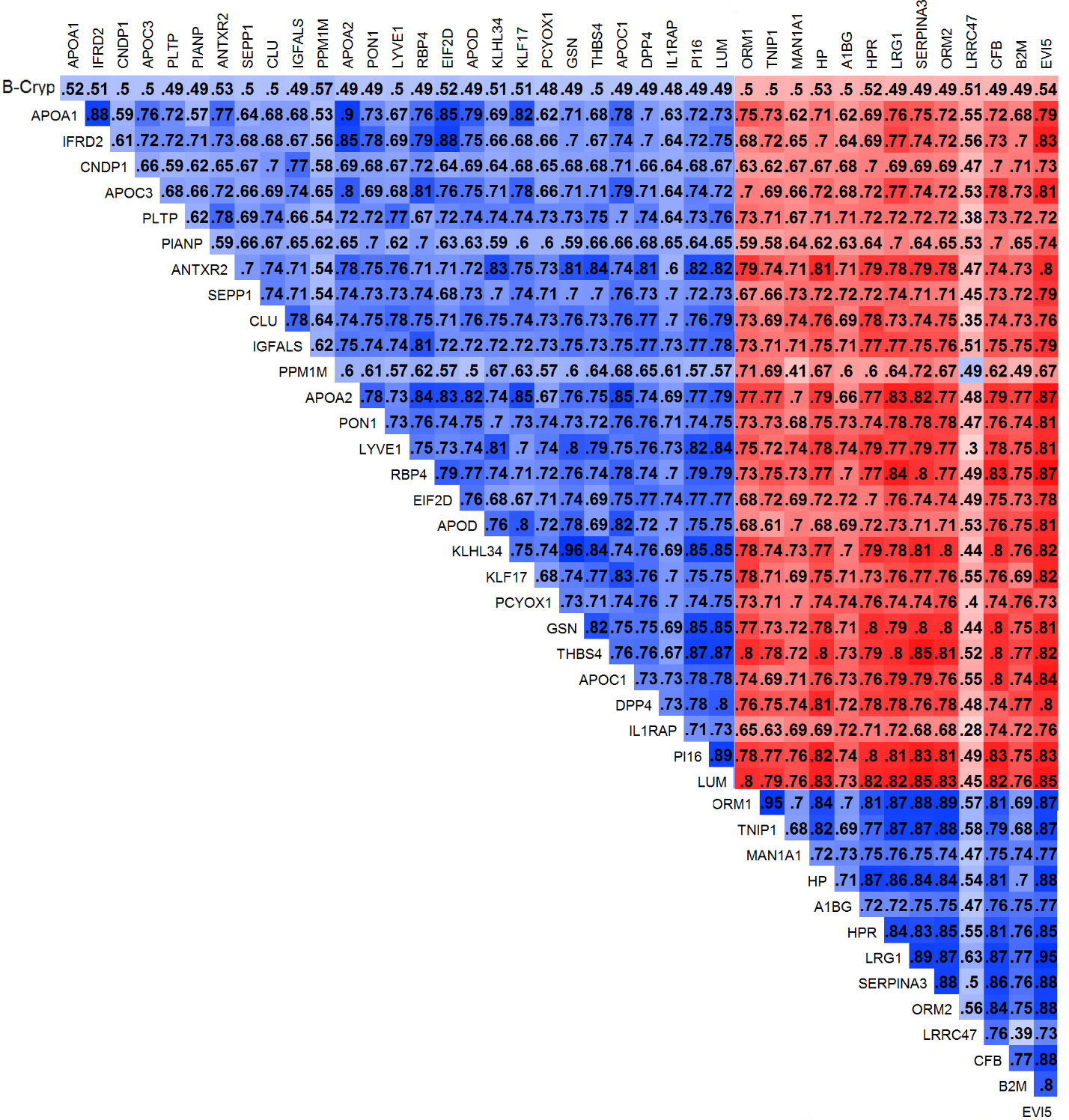


**Supplementary Table 2. Anthropometry, dietary intake, and morbidity patterns of Nepalese children, 6-8 years of age, in the plasma nutriproteomics study (n=500), Sarlahi, Nepal**

| Category | Values^1^ |
| --- | --- |
| Anthropometry^2^ |  |
| Weight, kg | 18.2 ± 2.2 |
| Height, cm | 114.1 ± 5.8 |
| Body mass index, kg/m^2^ | 14.0 ± 1.1 |
| WAZ^3^ | -1.99 ± 0.85 |
| HAZ^3^ | -1.77 ± 0.95 |
| BMIZ^3^ | -1.20 ± 0.89 |
| WAZ < -2^4^ | 48.5 |
| HAZ < -2^4^ | 39.1 |
| BMIZ <-2^4^ | 16.4 |
|  |  |
| Dietary intake, >3 times in past 7 days^5^, % |  |
| Dairy | 56.6 |
| Meat | 8.6 |
| Fish | 9.0 |
| Eggs | 2.2 |
| Dark green leaves | 32.4 |
| Yellow and Orange Fruits | 20.0 |
|  |  |
| Morbidity, any days in past week^6^ , % |  |
| Fever | 8.2 |
| Diarrhea | 3.2 |
| Productive cough | 3.8 |
| Rapid breathing and grunting | 2.8 |

^1^ Values are means ± SD, percentages.

^2^ n = 499

^3^ Weight-for-age z-score (WAZ), Height-for-age z-score (HAZ) and body mass index (BMI)-for-age z-score (BMIZ) based on World Health Organization reference distributions (32).

^4^ Percent <-2 z-score cutoffs reflect prevalence of underweight (WAZ), stunting (HAZ) and thinness (BMIZ) (32).

^5^ Dietary data are based on a parental 7-day food frequency without portion size assessment.

^6^ Morbidity assessment by 7-day parental history.

**REFERENCES**:

[1] Britton G, Liaaen-Jensen S, Pfander H. Carotenoids: Natural Functions. Vol. 4. Birkhäuser, Basel, Switzerland Springer Press, 2008.

[2] Lu S, Li L. Carotenoid metabolism: biosynthesis, regulation, and beyond. J. Integr. Plant Biol. 2008;50:778–85.

[3] Maresca JA, Graham JE, Bryant AD. The biochemical basis for structural diversity in the carotenoids of chlorophototrophic bacteria. Photosynth. Res. 2008;97:121–40.

[4] Takaichi S, Mochimaru M. Carotenoids and carotenogenesis in cyanobacteria: unique ketocarotenoids and carotenoid glycosides. Cell. Mol. Life Sci. 2007;64:2607–19.

[5] Ye ZW, Jiang JG, Wu GH. Biosynthesis and regulation of carotenoids in Dunaliella: progresses and prospects. Biotechnol. Adv. 2008;26:352–60.

[6] Romanchik JE, Morel DW, Harrison EH. Distributions of carotenoids and α-tocopherol among lipoproteins do not change when human plasma is incubated in vitro. J. Nutr. 1995;125:2610–17.

[7] Eroglu A, and Harrison EH. Carotenoid metabolism in mammals, including man: formation, occurrence, and function of apocarotenoids. Journal of Lipid Research 2013;54:1719–30.

[8] Narayanasamy S, Sun J, Pavlovicz ER, Eroglu A, Rush EC, Sunkel BD, Li C, Harrison, EH, and Curley RW. Synthesis and Biological Effects of Apo-11-, Apo-13-, and Apo-15-Lycopenoids, Cleavage Products of Lycopene that Function as Retinoic Acid Receptor Antagonists. Journal of Lipid Research 2017;58,1021–1029.

[9] Singh V, West KP Jr. Vitamin A deficiency and xerophthalmia among school-aged children in Southeastern Asia. Eur J Clin Nutr. 2004;58:1342–9.

[10] World Health Organization. Global prevalence of vitamin A deficiency in populations at risk 1995–2005. WHO Global database on vitamin A deficiency. Geneva, Switzerland: WHO; 2009.

[11] West KP Jr. Extent of vitamin A deficiency among preschool children and women of reproductive age. J Nutr. 2002;132:2857–66.

[12] Sommer A, West KP Jr. Vitamin A deficiency: health, survival and vision*.* Oxford University Press, New York. 1996.

[13] Burton GW, Ingold KU. beta-Carotene: an unusual type of lipid antioxidant. *Science*. 1984;224:569–73.

[14] Rubin L, Ross AC, Stephensen CB, Bohn T, Tanumihardjo SA. Metabolic Effects of Inflammation on Vitamin A and Carotenoids in Humans and Animal Models Adv Nutr. 2017;8:197–212

[15] Riccioni G, D’Orazio N, Speranza L, Di Ilio E, Glade M, Bucciarelli V, Scotti L, Martini F, Pennelli A, Bucciarelli T. Carotenoids and asymptomatic carotid atherosclerosis. J. Biol Regul Homeost Agents. 2010;24:447–52.

[16] Chatterjee M, Janarthan M. Biological activity of carotenoids: its implications in cancer risk and prevention. Curr Pharm Biotechnol. 2012;13:180–90.

[17] Zhou H, Zhao X, Johnson EJ, Lim A, Sun E, Yu J, Zhang Y, Liu X, Snellingen T, Shang F, Liu N. Serum carotenoids and risk of age-related macular degeneration in a Chinese population sample. Invest Ophthalmol Vis Sci. 2011;52:4338–44.

[18] Krinsky NI, Landrum JI, Bone RA. Biological mechanisms of the protective role of lutein and zeaxanthin in the eye. Ann. Rev. Nutr. 2003;23:171–201.

[19] Al-Delaimy WK, Slimani N, Ferrari P, Key T, Spencer E, Johansson I. Johansson G, Mattisson I, Wirfalt E, Sieri S. et al. Plasma carotenoids as biomarkers of intake of fruits and vegetables: ecological level correlations in the European Prospective Investigation into Cancer and Nutrition (EPIC). Eur J Clin Nutr 2005;59:1397–1408.

[20] McNaughton SA, Marks GC, Gaffney P, Williams G, Green A. Validation of a food-frequency questionnaire assessment of carotenoid and vitamin E intake using weighed food records and plasma biomarkers: the method of triads model. Eur J Clin Nutr 2005;59:211–218.

[21] Hodge AM, Simpson JA, Fridman M, Rowley K, English DR, Giles GG, Su Q, O'Dea K. Evaluation of an FFQ for assessment of antioxidant intake using plasma biomarkers in an ethnically diverse population. Public Health Nutr 2009;12:2438–47.

[22] Hess D, Keller HE, Oberlin B, Bonfanti R, Schüep W. Simultaneous determination of retinol, tocopherols, carotenes and lycopene in plasma by means of high-performance liquid-chromatography on reversed phase. Int J Vit Nutr Res 1991;61:232–8.

[23] Cole RN, Ruczinski I, Schulze K, Christian P, Herbrich S, Wu L, DeVine LR, O’Meally RN, Shrestha S, Boronina TN, et al. The plasma proteome identifies expected and novel proteins correlated with micronutrient status in undernourished Nepalese children. J Nutr 2013;143:1540–8.

[24] West KP Jr, Cole RN, Shrestha S, Schulze KJ, Lee SE, Betz J, Nonyane BA, Wu LS, Yager JD, Groopman JD, Christian P. A plasma alpha-tocopherome can be identified from proteins associated with vitamin E status in school-aged children of Nepal. J Nutr. 2015;12:2646–56.

[25] Schulze KJ, Cole RN, Chaerkady R, Wu LS, Nonyane BA, Lee SE, Yager JD, Groopman JD, Christian P, West KP Jr. Plasma selenium protein P isoform 1 (SEPP1): a predictor of selenium status in Nepalese children detected by plasma proteomics. Int J Vitam Nutr Res 2016 May 10 (Epub ahead of print; DOI: 10.1024/0300-9831/a000256).

[26] Lee SE, Schulze KJ, Wu LS, Yager JD, Christian P, West KP Jr. Biological Systems of Vitamin K: A Plasma Nutriproteomics Study of Subclinical Vitamin K Deficiency in 500 Nepalese Children. Omics 2016;4:214–23.

[27] Lee SE, West KP Jr, Cole RN, Schulze KJ, Christian P, Wu LS, Yager JD, Groopman JD, Ruczinki I. Plasma proteome biomarkers of inflammation in school aged children in Nepal. PLoS One 2015;10:e0144279 doi:10.1371/journal.pone.0144279 <http://dx.doi.org/10.1371/journal.pone.0144279>

[28] Lee SE, West KP Jr, Cole RN, Schulze KJ, Wu LS, Yager JD, Groopman JD, Christian P. General intelligence is associated with subclinical inflammation in Nepalese children: A population-based plasma proteomics study. Brain Behav Immun 2016;56:253–63.

[29] Lee SE, Stewart CP, Schulze KJ, Cole RN, Wu LS, Yager JD, Groopman JD, Khatry SK, Adhikari RK, Christian P, West KP Jr. The Plasma Proteome Is Associated with Anthropometric Status of Undernourished Nepalese School-Aged Children. J Nutr 2017;147:304–13.

[30] Christian P, Khatry SK, Katz J, Pradhan EK, LeClerq SC, Shrestha SR, Adhikari RK, Sommer A, West KP Jr. Effects of alternative maternal micronutrient supplements on low birth weight in rural Nepal: double blind randomized community trial. BMJ 2003;326:571–7.

[31] Schulze KJ, Christian P, Wu LS-F, Arguello M, Cui H, Nanayakkara-Bind A, Stewart C, Khatry SK, LeClerq S, West KP Jr. Micronutrient deficiencies are common in 6- to 8-year-old children of rural Nepal, with prevalence estimates modestly affected by inflammation. J Nutr 2014;144:979–87.

[32] WHO. Growth reference data for 5–19 years. Geneva: WHO; 2007 [cited 2012 Oct 11]. Available from: [**http://www.who.int/growthref/en/**](http://www.who.int/growthref/en/).

[33] Yamini S, West KP Jr., Wu L, Dreyfuss ML, Yang DX, Khatry SK. Circulating levels of retinol, tocopherol and carotenoid in Nepali pregnant and postpartum women following long-term beta-carotene and vitamin A supplementation. Eur J Clin Nutr 2001;55:252–9.

[34] Herbrich SM, Cole RN, West KP Jr., Schulze K, Yager JD, Groopman JD, Christian P, Wu L, O Meally RN, May DH, et al. Statistical inference from multiple iTRAQ experiments without using common reference standards. J Proteome Res 2013;12:594–604.

[35] Storey JD. A direct approach to false discovery rates. J R Stat Soc B 2002;64:479–98.

[36] Robinson GK. That BLUP is a good thing: the estimation of random

effects. Stat Sci. 1991;6:15–32.

[37] National Center for Biotechnology Information. Genetic sequence data bank [Internet]. Bethesda (MD): National Library of Medicine (US). c2016 [cited 2016 Oct 23]. Available from: [**ftp://ftp.ncbi.nih.gov/genbank/gbrel.txt**](ftp://ftp.ncbi.nih.gov/genbank/gbrel.txt).

[38] The Comprehensive R Archive Network [Internet]. Vienna (Austria): Institute for Statistics and Mathematics, Vienna University of Economics and Business. c2004 [cited 2015 May 27]. Available from: [**http://cran.r-project.org**](http://cran.r-project.org/)

[39] Beydoun, MA, Shroff, MR, Chen, X, Beydoun, HA, Wang, Y, Zonderman, AB. Serum antioxidant status is associated with metabolic syndrome among US adults in recent national surveys. J. Nutr. 2011;141:903–913.

[40] Shardell MD, Alley DE, Hicks, GE, El-Kamary, SS, Miller, RR, Semba, RD, Ferrucci, L. Low-serum carotenoid concentrations and carotenoid interactions predict mortality in US adults: The third national health and nutrition examination survey. Nutr. Res. 2011;31:178–189.

[41] Shah AS, Tan L, Lu Long J, Davidson WS. The proteomic diversity of high density lipoproteins: our emerging understanding of its importance in lipid transport and beyond. J Lipid Res. 2013;54: 2575–2585.

[42] Mangaraj M, Nanda R, Panda S. Apolipoprotein A-I: A Molecule of Diverse Function. Ind J Clin Biochem 2016;31:253–9.

[43] Enesa K, Moll HP, Luong L, Ferran C, Evans PC. A20 suppresses vascular inflammation by recruiting proinflammatory signaling molecules to intracellular aggresomes. FASEB J. 2015;29:1869–78.

# [44] Verstrepen L, Verhelst K, van Loo G, Carpentier I, Ley SC, Beyaert R. Expression, biological activities and mechanisms of action of A20 (TNFAIP3). Biochem Pharmacol. 2010;80;2009–20.

[45] Mayilyan KR. Complement genetics, deficiencies, and disease associations. Protein Cell 2012;3:487–96.

[46] Nielsen MJ, Moller HJ, Moestrup SK. Hemoglobin and heme scavenger receptors. Antioxid Redox Signal 2010;12:261–73.

[47] Nielsen MJ, Petersen SV, Jacobsen C, Oxvig C, Rees D, Moller HJ, Moestrup SK. Haptoglobin-related protein is a high-affinity hemoglobin-binding plasma protein. Blood. 2006;108:2846–9.

[48] Hochepied T, Berger FG, Baumannc H, Libert C. α1-Acid glycoprotein: an acute phase protein with inflammatory and immunomodulating properties. Cytokine Growth Factor Rev 2003;14:25–34.
[49] Kalsheker NA. Alpha-1-antichymotrypsin. Int J Biochem Cell Biol. 1996;28:961–64.

[50] Janciauskiene S. Conformational properties of serine proteinase inhibitors (serpins) confer multiple pathophysiological roles. Biochim Biophys Acta. 2001;1535:221–35.

[51] Piñeiro M, Andrés M, Iturralde M, et al. ITIH4 (inter-alpha-trypsin inhibitor heavy chain 4) is a new acute-phase protein isolated from cattle during experimental infection. Infect Immun. 2004;72:3777–82.

[52] Chisté RC, Freitas M, Mercadante AZ, Fernandes E. Carotenoids inhibit lipid peroxidation and hemoglobin oxidation, but not the depletion of glutathione induced by ROS in human erythrocytes Life Sci. 2014;99:52–60.

[53] Burk RF, Hill KE. Selenoprotein P-expression, functions, and roles in mammals. Biochim. Biophys. Acta 2009;1790:1441–47.

[54] Rotruck JT, Pope AL, Ganther HE, Swanson AB, Hafeman DG, Hoekstra WG. Selenium: biochemical role as a component of glutathione peroxidase. Science*.* 1973;179:588–90.

[55] Deakin S., Leviev I., Gomaraschi M., Calabresi L., Franceschini G., James R.W. Enzymatically active paraoxonase-1 is located at the external membrane of producing cells and released by a high affinity, saturable, desorption mechanism. J. Biol. Chem. 2002;277:4301–8.

[56] Bone RA, Landrum JT, Tarsis SL. Preliminary identification of the human macular pigment. Vision Res*.* 1985;25:1531–5.

[57] Barker FM, Snodderly DM, Johnson EJ, Schalch W, Koepcke W, Gerss J, Neuringer M. Nutritional manipulation of primate retinas, V: effects of lutein, zeaxanthin, and n-3 fatty acids on retinal sensitivity to blue-light-induced damage. Invest Ophthalmol Vis Sci. 2011;52:3934–3942.

[58] Krinsky NI, Johnson EJ. Carotenoid actions and their relation to health and disease. Mol. Aspects Med. 2205;26:459–516.

[59] Sasaki M, Ozawa Y, Kurihara T, et al. Neuroprotective effect of an antioxidant, lutein, during retinal inflammation. Invest Ophthalmol Vis Sci*.* 2009;50:1433–1439.

[60] Samson ML, Morrison S, Masala N, Sullivan BD, Sullivan DA, Sheardown H, Schmidt TA. Characterization of full-length recombinant human proteoglycan 4 as an ocular surface boundary lubricant. Exp Eye Res. 2014;127:14–19.

[61] Schmidt TA, Sullivan DA, Knop E, Richards SM, Knop N, Liu SH, Sahin A, Darabad RR, Morison S, Kam WR et al. Transcription, Translation, and Function of Proteoglycan 4, a Boundary Lubricant, at the Ocular Surface. Jama Ophthalmol. 2013;131:766–76.
